# High Zika Virus Seroprevalence in Salvador, Northeastern Brazil Limits the Potential for Further Outbreaks

**DOI:** 10.1128/mBio.01390-17

**Published:** 2017-11-14

**Authors:** Eduardo Martins Netto, Andres Moreira-Soto, Celia Pedroso, Christoph Höser, Sebastian Funk, Adam J. Kucharski, Alexandra Rockstroh, Beate M. Kümmerer, Gilmara Souza Sampaio, Estela Luz, Sara Nunes Vaz, Juarez Pereira Dias, Fernanda Anjos Bastos, Renata Cabral, Thomas Kistemann, Sebastian Ulbert, Xavier de Lamballerie, Thomas Jaenisch, Oliver J. Brady, Christian Drosten, Manoel Sarno, Carlos Brites, Jan Felix Drexler

**Affiliations:** a Hospital Universitário Professor Edgard Santos, Universidade Federal de Bahia, Salvador, Brazil; b Instituto Brasileiro para a Investigação da Tuberculose/Fundação José Silveira (IBIT/FJS), Salvador, Brazil; c Institute of Virology, University of Bonn Medical Centre, Bonn, Germany; d Institute for Hygiene and Public Health, GeoHealth Centre, WHO Collaborating Centre for Health Promoting Water Management & Risk Communication, University of Bonn, Bonn, Germany; e Centre for the Mathematical Modelling of Infectious Diseases, London School of Hygiene & Tropical Medicine, London, United Kingdom; f Department of Immunology, Fraunhofer Institute for Cell Therapy and Immunology, Leipzig, Germany; g German Centre for Infection Research (DZIF), Germany; h Maternidade Climério de Oliveira, Universidade Federal da Bahia, Salvador, Brazil; i Aix Marseille Université, IRD French Institute of Research for Development, EHESP French 19 School of Public Health, EPV UMR_D 190 “Emergence des Pathologies Virales,” Marseille, France; j IHU Institute hospitalo-universitaire Méditerranée Infection, APHM Public Hospitals of Marseille 21, Marseille, France; k Section Clinical Tropical Medicine, Department for Infectious Diseases, INF 324, Heidelberg University Hospital, Heidelberg, Germany; l Charité–Universitätsmedizin Berlin, corporate member of Freie Universität Berlin, Humboldt-Universität zu Berlin, and Berlin Institute of Health, Institute of Virology, Berlin, Germany; Centers for Disease Control and Prevention; Emory University School of Medicine

**Keywords:** Zika virus, microcephaly, risk factors, serology, socioeconomic status

## Abstract

During 2015 to 2016, Brazil reported more Zika virus (ZIKV) cases than any other country, yet population exposure remains unknown. Serological studies of ZIKV are hampered by cross-reactive immune responses against heterologous viruses. We conducted serosurveys for ZIKV, dengue virus (DENV), and Chikungunya virus (CHIKV) in 633 individuals prospectively sampled during 2015 to 2016, including microcephaly and non-microcephaly pregnancies, HIV-infected patients, tuberculosis patients, and university staff in Salvador in northeastern Brazil using enzyme-linked immunosorbent assays (ELISAs) and plaque reduction neutralization tests. Sera sampled retrospectively during 2013 to 2015 from 277 HIV-infected patients were used to assess the spread of ZIKV over time. Individuals were georeferenced, and sociodemographic indicators were compared between ZIKV-positive and -negative areas and areas with and without microcephaly cases. Epidemiological key parameters were modeled in a Bayesian framework. ZIKV seroprevalence increased rapidly during 2015 to 2016, reaching 63.3% by 2016 (95% confidence interval [CI], 59.4 to 66.8%), comparable to the seroprevalence of DENV (75.7%; CI, 69.4 to 81.1%) and higher than that of CHIKV (7.4%; CI, 5.6 to 9.8%). Of 19 microcephaly pregnancies, 94.7% showed ZIKV IgG antibodies, compared to 69.3% of 257 non-microcephaly pregnancies (*P* = 0.017). Analyses of sociodemographic data revealed a higher ZIKV burden in low socioeconomic status (SES) areas. High seroprevalence, combined with case data dynamics allowed estimates of the basic reproduction number *R*_0_ of 2.1 (CI, 1.8 to 2.5) at the onset of the outbreak and an effective reproductive number *R*_eff_ of <1 in subsequent years. Our data corroborate ZIKV-associated congenital disease and an association of low SES and ZIKV infection and suggest that population immunity caused cessation of the outbreak. Similar studies from other areas will be required to determine the fate of the American ZIKV outbreak.

## IMPORTANCE

The ongoing American Zika virus (ZIKV) outbreak involves millions of cases and has a major impact on maternal and child health. Knowledge of infection rates is crucial to project future epidemic patterns and determine the absolute risk of microcephaly upon maternal ZIKV infection during pregnancy. For unknown reasons, the vast majority of ZIKV-associated microcephaly cases are concentrated in northeastern Brazil. We analyzed different subpopulations from Salvador, a Brazilian metropolis representing one of the most affected areas during the American ZIKV outbreak. We demonstrate rapid spread of ZIKV in Salvador, Brazil, and infection rates exceeding 60%. We provide evidence for the link between ZIKV and microcephaly, report that ZIKV predominantly affects geographic areas with low socioeconomic status, and show that population immunity likely caused cessation of the outbreak. Our results enable stakeholders to identify target populations for vaccination and for trials on vaccine efficacy and allow refocusing of research efforts and intervention strategies.

## INTRODUCTION

During 2016, the Zika virus (ZIKV) outbreak in Latin America and the Caribbean was declared a public health emergency of international concern (1). Autochthonous circulation of ZIKV is now reported across vast areas of Latin America (2, 3).

Many countries in the Americas have reported high rates of clinically suspected ZIKV infections (2), but the proportion of laboratory-confirmed cases remains low. Case identification is hindered by the clinical similarities between ZIKV and endemic dengue virus (DENV) as well as Chikungunya virus (CHIKV) disease (4). Among the challenges in laboratory testing is the low and short-lived presence of ZIKV in body fluids (5). Furthermore, detection of ZIKV-specific antibodies in tropical regions is ambiguous due to cross-reactive antibodies elicited by previous infections with antigenically related viruses, including the widespread DENV (4), limiting accurate diagnostic testing even when using highly specific neutralization tests (6). In addition, asymptomatic courses in an estimated 80% of ZIKV-infected individuals (7) make clinical cases an insensitive measure of population-level exposure. Uncertainty about the ZIKV infection rate and proportion of the population exposed has key implications for modeling the trajectory of the American ZIKV outbreak (8, 9) and studies describing the etiology and frequency of ZIKV-associated congenital disease (10, 11).

**FIG 1:**
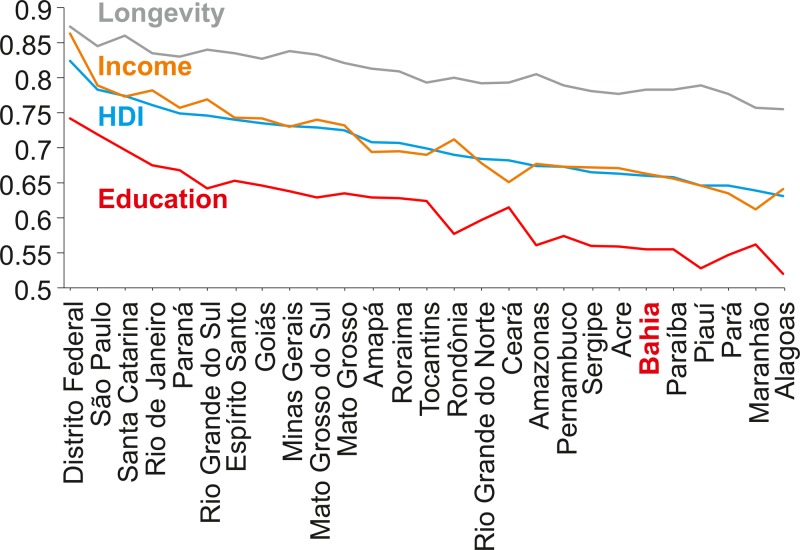
Ranking of Brazilian states according to the United Nations Development Programme. Longevity (gray), income (orange) and education (red) indexes, and the human development index (blue) as the geometric mean of the three aformentioned indexes. Data retrieved from Atlas Brazil, 2013 (http://www.atlasbrasil.org.br/2013/). The northeastern state Bahia is shown in bold and red.

For unknown reasons, northeastern Brazil has reported the vast majority of cases of ZIKV-associated microcephaly (12). Among the possible effect modifiers is the low socioeconomic status (SES) of the northeastern states of Brazil, exemplified by an approximately 5- to 10-fold lower monthly household income compared to more-affluent regions of Brazil (13). As shown in Fig. 1, the northeastern state of Bahia is one of the most underdeveloped Brazilian states according to the human development index (HDI) provided by the United Nations Development Programme (UNDP). Bahia was among the most ZIKV-affected regions in 2015 (14). However, the potential cofactors for ZIKV-associated microcephaly and whether these cofactors may be associated with low SES remain unclear.

Here, we investigate specimens sampled before, during, and after the current ZIKV outbreak to reconstruct the temporal spread of ZIKV in Salvador, the capital of Bahia, Brazil. We determine the infection rate of ZIKV in different subpopulations, explore its etiologic role in congenital disease, and use a mathematical modeling approach to project the trajectory of the ZIKV epidemic. Finally, we use a geographic information system-based approach to identify location-specific differences of ZIKV exposure and explore their associations with low SES.

## RESULTS

**FIG 2:**
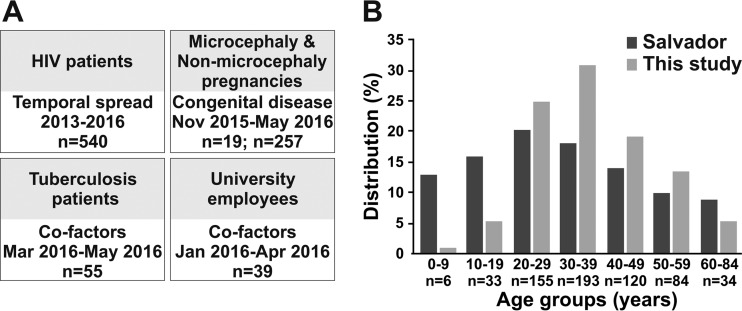
Serosurveys and distribution of specimens per age category. (A) Main research question, time span of sampling, and specimens per subpopulation. (B) Distribution of specimens per age category. Only specimens sampled for all subpopulations in 2015 to 2016 were included due to low Zika virus prevalence in the preceding years. The numbers (*n*) of study participants for which age information was available are given below the age categories. Age data for Salvador were retrieved from the 2010 census (https://cidades.ibge.gov.br/brasil/ba/salvador/panorama).

This study comprised 910 individuals from Salvador, Brazil, representing four different subpopulations. To assess the role of ZIKV in congenital disease, we collected specimens from parturients from 25 November 2015 to 2 May 2016. These specimens included samples from 16 mothers of neonates with microcephaly and three neonates with microcephaly for whom the mothers’ sera could not be obtained, as well as 255 mothers of neonates without microcephaly, including two neonates for whom the mothers’ sera could not be obtained. To investigate the temporal spread of ZIKV and to assess specificity of the serological tests, samples from 540 HIV-infected patients were used. These specimens included stored samples collected between 12 January 2013 to 30 August 2015 and samples from patients who attended HIV outpatient departments between 25 November 2015 to 28 May 2016. Finally, 55 tuberculosis patients and 39 university employees were sampled from 12 January 2016 to 28 May 2016 to investigate the impact of SES on ZIKV exposure (Fig. 2A). All adult age groups composing the general population of Salvador, Brazil, were represented in our study (Fig. 2B), and the subpopulations included in this study comprised individuals whose households were widely spread across urban Salvador (see Fig. S1 in the supplemental material). The main assay used for serological testing was a commercially available enzyme-linked immunosorbent assay (ELISA) relying on the recombinant NS1 antigen of ZIKV (15, 16), because this assay was the only test certified for serological diagnostics of ZIKV by the responsible Brazilian authority ANVISA (Agência Nacional de Vigilância Sanitária) and thus available to us during this study (17). Confirmatory testing conducted in about half of the sera used in this study included plaque reduction neutralization tests (PRNT) and an in-house ELISA relying on a recombinant envelope (E) antigen of ZIKV (56), designed to be robust against unspecific reactivity by targeted mutation of cross-reactive residues and preincubation of sera with heterologous antigens of the four DENV serotypes.

10.1128/mBio.01390-17.1 FIG S1  Spatial distribution of samples per subpopulation in Salvador, Bahia, Brazil. Panels A to D show the origin of samples for all subpopulations, with samples positive (red) and negative (blue) for antibodies against Zika virus (ZIKV) shown. Panels E and F show Chikungunya (CHIKV) and dengue (DENV) virus antibody-positive samples in red and different shapes, whereas antibody-negative samples of all subpopulations are shown in shades of blue for clarity of presentation. The orange star in panel E shows the single CHIKV antibody-positive microcephaly pregnancy. DENV includes only ZIKV antibody-negative specimens due to cross-reactivity of the DENV ELISA with ZIKV antibodies. HIV-infected patients from 2013 to 2015 are not shown due to low ZIKV positivity. Download FIG S1, PDF file, 19.5 MB. Copyright © 2017 Martins Netto et al. 2017 Martins Netto et al. This content is distributed under the terms of the Creative Commons Attribution 4.0 International license.

### ZIKV infection in parturients.

**FIG 3:**
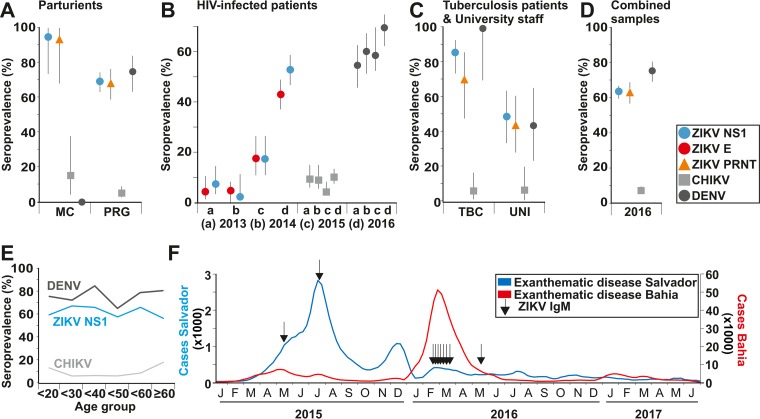
ZIKV seroprevalence and reported cases. (A) ZIKV, CHIKV, and DENV seroprevalence in parturients. Non-microcephaly pregnancies (PRG) (*n* = 257 for ZIKV IgG and CHIKV IgG and *n* = 69 for DENV IgG); microcephaly pregnancies (MC) (*n* = 19 for ZIKV IgG and CHIKV IgG and *n* = 0 for DENV IgG). (B) ZIKV, CHIKV, and DENV seroprevalence in HIV-positive patients from 2013 (*n* = 96 for ZIKV IgG and CHIKV IgG and *n* = 52 for DENV IgG), 2014 (*n* = 89 for ZIKV IgG and CHIKV IgG and *n* = 57 for DENV IgG), 2015 (*n* = 92 for ZIKV IgG and CHIKV IgG and *n* = 46 for DENV IgG), and 2016 (*n* = 263 for ZIKV IgG and CHIKV IgG and *n* = 110 for DENV IgG). (C) ZIKV, CHIKV, and DENV seroprevalence in tuberculosis patients (TBC) (*n* = 55 for ZIKV IgG and CHIKV IgG and *n* = 8 for DENV IgG) and university employees (UNI) (*n* = 39 for ZIKV IgG and CHIKV IgG and *n* = 20 for DENV IgG). (D) ZIKV, CHIKV, and DENV seroprevalence in all 633 samples from 2016. The bars in panels A to D depict 95% confidence intervals. (E) Seroprevalence per age group for ZIKV IgG, CHIKV, and DENV in 633 samples from 2016. (F) Reported Brazilian cases of acute exanthematic disease in Salvador and Bahia until epidemiological week 22 in 2017. The months are indicated by capital first letter.

A case-control study conducted in the neighboring northeastern metropolis Recife, Brazil, suggested an etiologic role of ZIKV in congenital disease (18). Consistent with these data, 18 of 19 parturients whose neonates were born with microcephaly (termed microcephaly pregnancies) from Salvador, Brazil, showed IgG antibodies against ZIKV (94.7%; 95% confidence interval [CI], 73.5 to 99.9%), compared to 69.3% of 257 non-microcephaly pregnancies using an NS1-based ELISA (CI, 63.3 to 74.5%; Table 1 and Fig. 3A). The higher ZIKV seroprevalence in microcephaly pregnancies compared to non-microcephaly pregnancies was statistically significant (*P* = 0.017 by Fisher’s exact test; relative risk = 1.4 [CI, 1.2 to 1.6]) and similar to ZIKV infection in 80.0% of microcephaly pregnancies compared to 63.9% of controls in Recife (18). Data from PRNT and the NS1 antigen ELISA were highly consistent (Table 1 and Fig. 3A). Unfortunately, lack of adequate sera taken close to birth prevented determination of ZIKV-specific IgM in all newborns with microcephaly.

**TABLE 1:** Serological test results^a^

Subpopulation^b^	Median age (yr) (IQR)^c^	Total no. of individuals tested for ZIKV by ELISA	ZIKV IgM		ZIKV IgG		ZIKV PRNT		CHIKV IgG		DENV IgG^d^	
			*n*	%	*n*	%	*n*/total no.	%	*n*/total no.	%	*n*/total no.	%
HIV patients												
2013	36.7 (16.4)	96	0	0	7	7.3			7/96	7.3	52/84	61.9
2014	38.8 (17.8)	89	0	0	2	2.3			6/89	6.7	57/82	69.5
2015	36.6 (17.4)	92	2	2.2	16	17.4			1/92	1.1	46/68	67.6
Total retrospective		277										

HIV patients 2016	44.7 (15.4)	263	2	0.8	139	52.9	31/61	50.8	22/263	8.4	88/110	80.4
MC pregnancies 2015–2016	28.5 (10.8)	19	1	5.3	18	94.7	14/15	93.3	3/19	15.8	0/1	0
Non-MC pregnancies 2015–2016	28.9 (10.9)	257	1	0.4	178	69.3	114/171	66.6	15/257	5.8	52/69	75.4
Tuberculosis patients 2016	45.1 (22.2)	55	2	3.6	47	85.5	14/20	70	4/55	7.3	8/8	100
University employees 2016	33.8 (12.3)	39	2	5.1	19	48.7	14/32	43.8	3/39	7.7	8/18	44.4
Total 2015–2016		633	8	1.3	401	63.3	187/299	62.5	47/633	7.4	156/206	75.7
Total study		910										

^a^ The number of specimens (*n*) and percentage of specimens positive for antibodies against Zika (ZIKV), Chikungunya (CHIKV), or dengue (DENV) virus in ELISA or plaque-reduction neutralization test (PRNT) are shown.

^b^ MC, microcephaly.

^c^ Interquartile range (IQR) shown in parentheses in the table.

^d^ Including only ZIKV-negative specimens due to cross-reactivity of the DENV ELISA with ZIKV antibodies.

### Temporal spread of ZIKV.

Phylogenetic reconstructions have suggested that ZIKV was introduced into the Americas during mid-late 2013 (14, 19). To assess whether the projected time of introduction can be confirmed by population-level antibody responses, we tested specimens from HIV-infected patients collected between 2013 and 2016. Retrospective specimens were available from routine attendance of HIV-infected patients for viral load measurements and resistance genotyping within the Brazilian HIV treatment program. Unfortunately, DENV-specific antibodies can cause false-positive ZIKV test results even when using highly specific PRNTs (20). Comparison of titer magnitudes between DENV and ZIKV PRNTs may support virological diagnostics of ZIKV exposure in paired sera from cases of acute febrile illness. However, ZIKV and DENV PRNT titers can range from 1:10 to about 1:100,000 in secondary flavivirus infections (20). DENV PRNTs are thus not an optimal solution to distinguish ZIKV from DENV exposure in a population-based sample from an area that is hyperendemic for DENV. Therefore, the sera from HIV-infected patients collected over 4 years were tested for ZIKV-specific IgG using an NS1 antigen ELISA and in parallel an E-antigen competitive ELISA. Both ELISAs yielded highly congruent results (Fig. 3B). ZIKV IgG seroprevalence increased from 4.2 to 7.3% in 2013 to 2014 (CI, 1.3 to 9.1%) to 17.4% in 2015 (CI, 10.9 to 26.5%) and to 43.0 to 52.9% in 2016 (CI, 37.1 to 58.8%; Fig. 3B and Table S1). The significant increase in seroprevalence (χ^2^ = 127.7 and *P* < 0.001 with the NS1 antigen ELISA and χ^2^ = 90.6 and *P* < 0.001 with the E-antigen competitive ELISA) corroborated the fast ZIKV spread in Salvador, Brazil, during 2015 to 2016 and suggested the reliability of both ELISAs in an area that is hyperendemic for DENV, as illustrated by 61.9 to 80.4% of sera reactive for DENV during 2013 to 2016 (Fig. 3B and Table 1). The significantly lower numbers of ZIKV IgG detections in 2013 to 2014 may correspond to the initial phase of ZIKV introduction into Salvador.

10.1128/mBio.01390-17.4 TABLE S1  Zika virus test results in HIV-infected patients. Results of NS1-based ELISA, E-protein-based competitive ELISA, and plaque reduction neutralization tests (PRNT) in HIV-infected patients sampled during 2013 to 2016 Download TABLE S1, DOCX file, 0.01 MB. Copyright © 2017 Martins Netto et al. 2017 Martins Netto et al. This content is distributed under the terms of the Creative Commons Attribution 4.0 International license.

### Patterns of ZIKV spread in Salvador, Brazil.

In northeastern Brazil, low socioeconomic conditions are major determinants of developing tuberculosis (21). To obtain preliminary evidence for ZIKV infection rates in different social strata within Salvador, Brazil, we therefore analyzed 55 low-SES patients treated for active tuberculosis (did not graduate from college, most patients without complete secondary schooling) and 39 healthy university employees (most with college education, all completed secondary schooling). As shown in Fig. 3C, significantly more tuberculosis patients (85.5%; CI, 73.6 to 92.7%) than university employees (48.7%; CI, 33.9 to 63.8%) showed ZIKV-specific antibodies (χ^2^ = 14.7; *P* = 0.0001) using the NS1 antigen ELISA. When only PRNT results were considered, the difference in seroprevalence between these two groups was similar to that of the NS1-based analysis and statistically significant, albeit at a lower significance level (χ^2^ = 4.48; *P* = 0.044). Similar to a study demonstrating higher DENV exposure in low-SES strata of the neighboring northeastern metropolis Recife prior to the introduction of ZIKV (22), DENV seroprevalence was significantly higher in tuberculosis patients at 100% (CI, 70.7 to 100%) than in university employees at 44.4% (CI, 24.5 to 66.3%; χ^2^ = 7.22; *P* = 0.007). This suggested more common exposure to arboviruses in low-SES strata in Salvador and validity of the comparison of ZIKV exposure in these subpopulations as proxy variables for different SESs.

Combining all study groups, ZIKV seroprevalence in Salvador, Brazil, in 2016 was 63.3% (CI, 59.4 to 66.8%) according to an NS1 antigen ELISA and 62.5% (CI, 56.9 to 67.8%) according to PRNT. Seroprevalence estimates according to the NS1 antigen ELISA and PRNT were thus near-identical (Fig. 3D and Table 1), even though NS1 antigen ELISA and PRNT results varied in 14.7% of individual specimens (Table 2). Despite its recent introduction, the seroprevalence of ZIKV thus almost reached that of the endemic DENV at 75.7% (CI, 69.4 to 81.1%), although DENV seroprevalence was still significantly higher (χ^2^ = 10.1; *P* = 0.001; Fig. S1 and Table 1). The high DENV seroprevalence in Salvador, Brazil, corresponded to a previous study reporting around 80 to 90% population-level DENV seroprevalence in northeastern Brazil before the introduction of ZIKV (22). No significant differences in ZIKV seroprevalence between male and female study participants were observed within subpopulations (Table S2). Finally, all age groups in this study showed similar ZIKV antibody detection rates (χ^2^ = 6.6; *P* = 0.4; Fig. 3E and Table S3), suggesting widespread rapid transmission with no age-related variation in exposure. These data suggested rapid spread of ZIKV within Salvador and were consistent with the age distribution observed during the 2007 Yap ZIKV outbreak (4).

**TABLE 2:** ZIKV NS1-based ELISA performance in prospectively collected specimens^a^

Subpopulation	No. of specimens within subpopulations	No. of specimens tested by PRNT (%)	ELISA result	No. of specimens with the following PRNT result:		% specimens with divergent ELISA/PRNT results	Sensitivity (95% CI)	Specificity (95% CI)	PPV (95% CI)	NPV (95% CI)
				+	−					
MC pregnancies	19	15 (78.4)	+	14	0	0	1 (0.76−1)	1 (0.25−1)	1 (0.76−1)	1 (0.25−1)
			−	0	1	0				
Non-MC pregnancies	257	171 (66.5)	+	105	21	12.3	0.92* (0.85−0.96)	0.63* (0.49−0.75)	0.83* (0.76−0.89)	0.80* (0.65−0.90)
			−	9	36	5.3				
HIV patients	263	61 (23.2)	+	26	6	9.8	0.83* (0.66−0.95)	0.80* (0.63−0.92)	0.81* (0.65−0.93)	0.83* (0.64−0.94)
			−	5	24	8.2				
Tuberculosis patients	55	20 (36.4)	+	14	0	0	1* (0.76−1)	1* (0.54−1)	1* (0.76−1)	1* (0.54−1)
			−	0	6	0				
University employees	39	32 (82.1)	+	12	1	3.1	0.86* (0.57−0.98)	0.94* (0.73−0.99)	0.92* (0.64−0.99)	0.89* (0.67−0.99)
			−	2	17	6.3				
Total specimens	633	299 (47.2)	+	171	28	9.4	0.91* (0.86−0.95)	0.75* (0.66−0.83)	0.86* (0.80−0.91)	0.84* (0.75−0.90)
			−	16	84	5.4				

^a^ The positive (+) and negative (−) ELISA and plaque reduction neutralization test (PRNT) results are shown. The sensitivity, specificity, positive predictive value (PPV), and negative predictive value (NPV), and adjusted Wald confidence intervals (95% CI) are shown; values with an *P* value of <0.0001 are indicated by an asterisk. MC, microcephaly.

10.1128/mBio.01390-17.5 TABLE S2  Gender distribution in prospectively sampled subpopulations. The proportions of male and female individuals and results of serological tests for Zika virus within subpopulations are shown. Download TABLE S2, DOCX file, 0.02 MB. Copyright © 2017 Martins Netto et al. 2017 Martins Netto et al. This content is distributed under the terms of the Creative Commons Attribution 4.0 International license.

10.1128/mBio.01390-17.6 TABLE S3  Age distribution of study participants compared to the population of Salvador, Brazil. The proportions of participants in different age groups comprised in this study compared to the proportion of the same age groups within the general population of Salvador, as well as the results of serological tests for Zika virus, dengue virus, and Chikungunya virus per age group are shown. Download TABLE S3, DOCX file, 0.02 MB. Copyright © 2017 Martins Netto et al. 2017 Martins Netto et al. This content is distributed under the terms of the Creative Commons Attribution 4.0 International license.

### Differential spread of ZIKV and CHIKV.

The emergence of CHIKV in the Americas parallels that of ZIKV spatiotemporally with the introduction and transcontinental spread in 2013 to 2014, and both viruses use *Aedes* mosquitos as vectors (23–25). However, CHIKV seroprevalence remained consistently low in HIV patients during 2013 to 2016 at 1.1 to 8.4% (χ^2^ = 5.9; *P* = 0.12; Fig. 3B, light gray), reaching an overall seroprevalence of 7.4% across all study groups in 2016 (CI, 5.6 to 9.8%; Fig. 3D and Table 1). Our CHIKV seroprevalence estimate was consistent with that of an independent study from Salvador, Brazil (26). Our data thus suggested an accelerated dissemination of ZIKV compared to CHIKV in Salvador, Brazil.

### Low rate of acute ZIKV infections in 2016.

Cases of acute exanthematic disease, the lead symptom of ZIKV infection in adults, reported in the Bahia state within the Brazilian surveillance system SINAN were retrieved and compared to our observations. Of the 67,454 cases reported in Bahia during 2015, 35,261 originated from Salvador (52.3%) (Fig. 3F, blue line). The first year of the ZIKV outbreak in Bahia was thus concentrated in Salvador. In contrast, in 2016, 59,054 ZIKV cases were reported all over Bahia (Fig. 3F, red line) of which only 928 cases originated from Salvador (1.6%). To see whether the decline of notified cases from Salvador in 2016 could be confirmed by laboratory tests, we tested all specimens for ZIKV RNA and ZIKV IgM. None of the specimens tested positive for ZIKV RNA. In agreement with the high ZIKV seroprevalence from 2015 onwards, IgM-based incidence was detected only in 2015 (2.2%; CI, 0.1 to 8.0%) and 2016 (1.3%; CI, 0.6 to 2.5%) (Fig. 3F, arrows). Until mid-2017, the number of reported cases remained consistently low from both Salvador and the state of Bahia. This suggests that the outbreak ceased due to the lack of acute cases.

### Modelling the trajectory of the epidemic in Salvador, Brazil.

**FIG 4:**
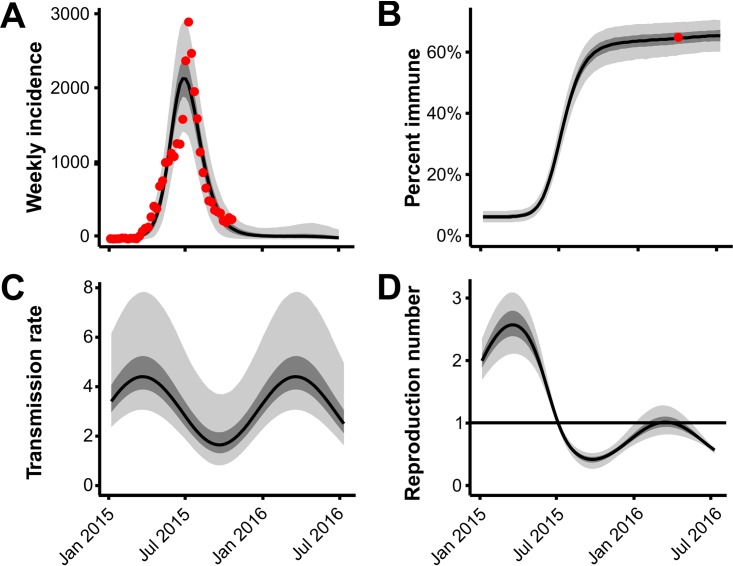
Transmission model and projected trajectory of the Zika epidemic in Salvador, Brazil. (A) Model fit to ZIKV incidence in Salvador. The red circles show the reported ZIKV cases. The black line shows the median model estimate. The shaded regions depict the interquartile range and 95% CI. (B) ZIKV seroprevalence over time in the study population (*n* = 633). The black line shows the median model estimate. The shaded regions depict the interquartile range (IQR) and 95% CI. The red circle shows the observed proportion of seropositive individuals. (C) Estimated seasonal variation in ZIKV transmission. (D) Estimated change in effective reproduction number over time.

To test whether population immunity would limit future cases in Salvador, Brazil, we fitted a mathematical model of ZIKV transmission jointly to the independent case notification data from Salvador and the seroprevalence results from our study. Our results showed that the observed data are consistent with a single-year continuous epidemic that began early in 2015 and declined toward the end of 2015 (Fig. 4A and B).

The estimated basic reproduction number (*R*_0_) for ZIKV was 2.1 (CI, 1.8 to 2.5) at the onset of the outbreak with, on average, 2.0% (CI, 1.8 to 2.2%) of ZIKV infections reported in the national surveillance system. Projecting the model forward into 2016 suggested a continued decline in transmission despite the return of peak arbovirus season. Due to the lack of susceptible individuals, the effective reproductive number (*R*_eff_) was not predicted to exceed one in subsequent years, a condition required for another ZIKV epidemic wave (Fig. 4C and D).

### Impact of SES.

**FIG 5:**
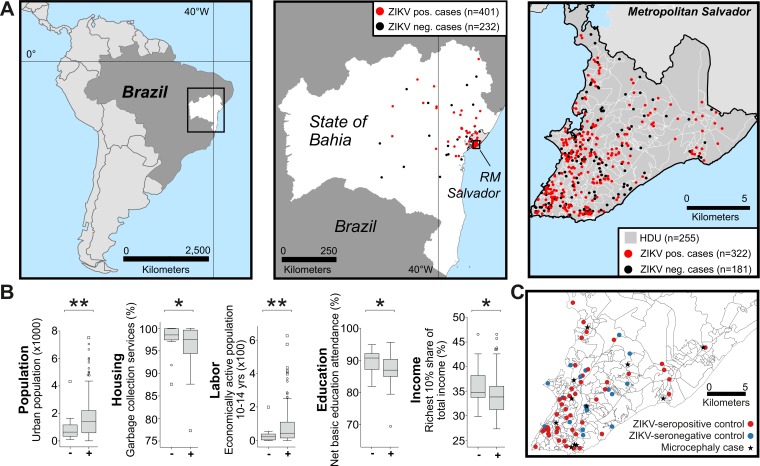
Association of socioeconomic status and ZIKV exposure. (A) Maps showing Brazil, the state of Bahia, metropolitan Salvador, and sample distribution onto human development units (HDUs). (B) Sociodemographic indicators differing significantly between ZIKV-positive and ZIKV-negative HDUs. Boxplots show medians, interquartile range (box length), outliers (circles), and extreme values (squares). Values that are significantly different are indicated by bars and asterisks as follows: *, *P* ≤ 0.05; **, *P* < 0.01. (C) Distribution of samples used for nested case-control study. ZIKV-positive and -negative cases and microcephaly pregnancies (stars; *n* = 11) are shown. One additional case was outside the area shown in the map. Seven other cases were insufficiently georeferenced. Due to geographic proximity of home adresses of some controls, not all 72 controls are visible.

To further investigate the impact of low SES on ZIKV infection rates, the home addresses of study participants were georeferenced onto 147 spatial units classified into human development units (HDUs) according to sociodemographic characteristics (Fig. 5A and Fig. S1). HDUs showing ZIKV-positive cases represented significantly lower SES in 65 (32.2%) of 201 indicators (Table S4). The latter included all 56 available population indicators, as well as less regular garbage recollection, a higher proportion of child and youth labor, inferior schooling, and lower income in ZIKV-positive HDUs (Fig. 5B shows the most significant indicators per category). No significant differences were observed regarding the occurrence of microcephaly in HDUs. Logistic regression analyses were conducted to identify which SES-associated indicators were most associated with ZIKV-positive HDUs. However, the high degree of multicollinearity between sociodemographic indicators prevented model convergence.

10.1128/mBio.01390-17.7 TABLE S4  Association between ZIKV infections and sociodemographic indicators. Comparison of sociodemographic indicators between those human development units (HDU) comprising households of individuals testing positive and those HDUs comprising households of individuals testing negative for Zika virus infection in a serological assay (NS1 antigen ELISA). Download TABLE S4, DOCX file, 0.05 MB. Copyright © 2017 Martins Netto et al. 2017 Martins Netto et al. This content is distributed under the terms of the Creative Commons Attribution 4.0 International license.

Finally, a nested case-control approach was conducted to investigate whether low-SES-associated indicators influenced the occurrence of microcephaly independently of ZIKV infection. To that end, six pregnant women matched for age (within 2 years) for each of 12 microcephaly cases living in HDUs within metropolitan Salvador, Brazil, were chosen, and the sociodemographic indicators of the respective HDUs were attributed to cases and controls (Fig. 5C). Only the ZIKV serostatus differed significantly between cases and controls (χ^2^ = 4.1; *P* = 0.043), in contrast to the sociodemographic indicators.

## DISCUSSION

Here we present the results of what is, to the best of our knowledge, the first arboviral seroprevalence survey in Latin America since the beginning of the Zika epidemic. We demonstrate a high ZIKV infection rate of about 63% in Salvador, the third-largest Brazilian city with about 2.7 million inhabitants in northeastern Brazil. This rate was comparable to the 66 to 73% seroprevalence found on Yap, Micronesia, and French Polynesia, although these ZIKV outbreaks occurred in 10- to 300-fold smaller island populations (10, 27). The similar seroprevalence rates suggest effective ZIKV spread irrespective of different geographic settings.

The reasons for the differential spread of ZIKV and CHIKV in Salvador, Brazil, remain unclear. Hypothetically, the faster spread of ZIKV might be associated with viral properties affecting transmission. However, a putative replicative advantage of ZIKV over CHIKV in Brazilian *Aedes* mosquitos is not warranted by vector competence studies (28, 29). Similarly, increased availability of ZIKV to mosquito vectors during feeding on viremic humans is unlikely, since viral loads can be considerably higher in CHIKV infections than in ZIKV infections (5). An alternative explanation may include amplification of CHIKV in sylvatic cycles prior to its putative introduction into urban cycles in Salvador, Brazil. However, whether CHIKV may enter a sylvatic cycle in the Americas remains to be determined (30). Finally, whereas sexual transmission of ZIKV may have contributed to its initial spread, the predominant route of transmission likely remains vector-borne, opposing a relatively faster spread of ZIKV due to sexual transmission (31). So far, the most plausible explanation may include differences in the geospatial introduction of CHIKV and ZIKV within northeastern Brazil. Indeed, the main foci of CHIKV infections in the Brazilian state of Bahia were initially centered in the hinterland, whereas ZIKV may have been directly introduced to the densely populated Atlantic coast, including Salvador, facilitating efficient spread in relatively larger, more connected human populations (32, 33).

Our modeling estimates of the basic reproduction number *R*_0_ were lower than in estimates for Pacific island populations (34) but consistent with recent estimates from several independent studies (8, 31, 35). Moreover, our data and modeling projections suggest that ZIKV was able to reach the critical population immunity threshold within a single year and that community protective immunity could restrict ZIKV spread in this area until susceptible individuals are replaced by birth or migration. This finding is consistent with the near-complete lack of reported cases from Salvador, Brazil, since 2016 and with previous model-based projections that predicted the cessation of the current Latin American outbreak within the next few years (8). The limitation of ZIKV spread due to community protective immunity is probably analogous to CHIKV, because both viruses show limited antigenic variability. Consistent with our data, CHIKV infection rates exceeding 60% have been associated with the cessation of outbreak activity (36). In Africa and probably in Asia as well, CHIKV can emerge cyclically from nonhuman primate reservoirs upon replenishment of sufficient numbers of susceptible individuals (36). Whether ZIKV can establish a sylvatic transmission cycle in Latin America thus requires urgent investigation (37).

The high rate of ZIKV-positive mothers of microcephaly cases in our study substantiates the recent case-control study from Recife, Brazil (18) in identifying ZIKV as the cause of the surge in microcephaly cases in northeastern Brazil. Additionally, our data enable more precise risk estimates of congenital ZIKV disease. In the absence of serological data, the risk of fetal microcephaly upon maternal ZIKV infection in the first trimester has previously been modeled across a seroprevalence interval spanning 10 to 80% (10). According to that study (10), the 63% seroprevalence rate found in this study implies a risk of fetal microcephaly in Bahia of about 1% during the first trimester. This risk is analogous to the 0.95% risk modeled for French Polynesia assuming a similar ZIKV infection rate of 66% (27) and similar to the 1.7% prevalence of microcephaly found in ZIKV-infected mothers in a cohort study in French Guiana (38).

Finally, our results suggest an impact of low SES on the probability of ZIKV infection. Whether the increased ZIKV infection rate correlates with increased risk of microcephaly remains to be determined, but it is in line with anecdotal evidence from the Brazilian Ministry of Health (39). Our data correspond to a previous study demonstrating higher DENV infection rates in lower social strata from northeastern Brazil (22). However, other etiologic factors associated with low SES remain to be determined in large prospective epidemiological studies, including detailed assessments of individual-level determinants of SES, exhaustive assessments of infectious and noninfectious causes of congenital malformations, clinical symptoms other than microcephaly, and differences in access to abortion practice between different social strata in Latin America, which may cause a relatively higher incidence of neonates with malformations in lower social strata because higher social strata may have a relatively easier access to antenatal care, including imaging techniques allowing premature identification of malformations leading to abortion practices (40–42). Of note, our data may imply that individuals and areas with a relatively higher SES may represent a potential reservoir for focal reemergence of ZIKV in Salvador, Brazil. However, whether high-SES strata may represent a sufficient community size to allow ZIKV resurgence in Salvador remains to be determined.

The strengths of our study include the large sample from different subpopulations that can identify key variations in transmission rates, the longitudinal analysis of patients before, during, and after the Zika outbreak, the multidisciplinary approach allowing insights into geospatial and sociodemographic factors affecting ZIKV exposure, and the comparison of seroprevalence of multiple arboviruses using a range of laboratory tests. A principal limitation of this study is the availability-based sample of individuals which may not be representative of the general population. However, the age distribution of individuals across the pooled samples was comparable to that of the general population, and infection rates in pregnant women were comparable to the overall seroprevalence from the combined subpopulations. Finally, seroprevalence results were comparable to (i) the independent case data from Salvador, Brazil, (ii) previous ZIKV seroprevalence surveys in other areas, and (iii) the seroprevalence results for DENV and CHIKV in other settings, suggesting that our study is robust despite our nonsystematic sampling design. Importantly, our seroprevalence data enabled an estimate of *R*_0_ that was highly consistent with estimates from other studies not containing serological information from the current American outbreak (8, 31, 35). The similarities between those modeling approaches and our data were thus supportive of the appropriateness of our data set. However, a principal challenge to our study arises from the high levels of cross-reactivity of antibodies elicited by different flaviviruses in serological tests, limiting the ability to obtain unequivocal serological results (43). Previous studies assessing the specificity of the NS1-based ELISA we used in our study yielded conflicting results (15, 44). However, the majority of studies aiming at test validation investigated patients with acute febrile illness and included only a few or no sera from individuals living in areas where DENV is endemic, limiting the ability to extrapolate results from those studies to our study population. Recent studies investigating asymptomatic blood donors from Martinique and Cameroon suggested applicability of the NS1-based ELISA, despite a high DENV burden in these areas (45, 46). Furthermore, our NS1-based ELISA results were largely congruent with PRNT-based analyses conducted within subpopulations. Of note, recent data suggest that PRNT specificity in late convalescent-phase sera may be high enough to retain its utility as a tool for population-level ZIKV serosurveillance (47). In sum, our seroprevalence data for samples collected during four consecutive years, before and during the dissemination of ZIKV in Salvador, Brazil, using two different ZIKV antigens for ELISA, and confirmation of ELISA results by PRNT strongly suggest that our data are valid despite the limitations of any serological investigation of ZIKV-specific antibody responses in areas in which other flaviviruses are hyperendemic. Of note, applicability of the NS1-based ELISA in our population-based study does not translate into a recommendation of its usage for patient diagnostics, which may require further validation and innovative tools that are not yet broadly available, such as a recently published monoclonal antibody-based competitive ELISA (48).

In summary, our data demonstrate high ZIKV infection rates in a Brazilian setting and suggest that the ZIKV outbreak ceased due to community protective immunity. Prevention of congenital ZIKV disease may need to incorporate responses to low SES-associated cofactors in addition to pathogen-oriented measures. Further studies of outbreak settings are urgently needed outside northeastern Brazil to determine whether such explosive and underrecognized ZIKV epidemics have also occurred. Ideally, these studies should include sera from neonates with congenital disease and their mothers sampled early during pregnancy, as well as specimens from adults suffering from severe ZIKV disease to identify whether determinants of severe ZIKV disease are shared among congenital and adult infections.

## MATERIALS AND METHODS

### Ethical clearance, sampling sites, and sample storage.

Sampling and testing were approved by the Federal University of Bahia (UFBA) research ethics board Climério de Oliveira under protocol 1.408.499. HIV patients were sampled at the UFBA teaching hospital. Tuberculosis patients were sampled at the José Silveira Foundation-Brazilian Institute for Investigation of Tuberculosis. Pregnant women were sampled at the time of delivery at the UFBA maternity hospital Climério de Oliveira. All patients attended during the study period accepted participation in the protocol. Microcephaly was diagnosed when the measurement of the cephalic circumference was 2 standard deviations below that of the corresponding gestational age, based on intergrowth charts from the World Health Organization in addition to clinical and imaging data as recommended (49).

### Laboratory analyses.

All samples were analyzed for viral RNA using real-time reverse transcription-PCR (RT-PCR) assays for ZIKV (5). Serological testing was performed by using enzyme-linked immunosorbent assays (ELISAs) for ZIKV IgM/IgG (Euroimmun, Lübeck, Germany) (NS1 antigen), DENV IgG (Euroimmun) (full virus lysates), and CHIKV IgG (Euroimmun) (recombinant structural protein) according to the manufacturer’s instructions. Briefly, sera diluted 1:101 in sample buffer were added to the wells and allowed to react for 60 min at 37°C. Before IgM detection, sera were preincubated with sample buffer containing IgG/rheumatoid factor absorbent (Euroimmun) to remove class IgG antibodies. Bound antibodies were detected by applying goat anti-human IgM peroxidase conjugate or rabbit anti-human IgG peroxidase conjugate for 30 min at room temperature. The competitive ELISA using a mutant E protein of ZIKV was conducted according to reference 50 for DENV and is described in detail elsewhere (56). Briefly, the quadruple mutant E protein from ZIKV (strain H/PF/2013, E-protein amino acid residues 1 to 406, GenBank accession no. KJ776791) bearing the point mutations T76A, Q77G, W101R, and L107R was expressed in *Drosophila* S2 cells. Serum samples (diluted 1:100 in 100 µl blocking solution) were preincubated with 2 µg/sample of mutant DENV E proteins (mixture of the four DENV serotypes [50] for 1 h to remove DENV antibodies and/or cross-reacting antibodies). Following the preincubations, samples were transferred to 96-well plates coated overnight with ZIKV mutant E protein (150 ng/well), and the assay was completed following standard ELISA procedures.

Due to 100% cross-reactivity of ZIKV-specific IgG antibodies with the DENV ELISA antigen (43), only clearly ZIKV-negative specimens were used for assessments of DENV seroprevalence (Fig. S2). Plaque reduction neutralization tests (PRNTs) for ZIKV (51) were used for confirmation in 199 ELISA-positive specimens and 100 ELISA-negative specimens from 2016 for which sufficient serum volumes were available (Table 2). All sera were heat inactivated (56°C, 30 min) prior to neutralization testing. Two microliters of serum was diluted in 1% Dulbecco modified Eagle medium (DMEM) at 1:25, 1:250, 1:2,500, and 1:25,000 and incubated at 37°C for 60 minutes with 50 plaque-forming units (PFU) of ZIKV outbreak strain H/PF/2013 resurrected from subgenomic cDNA fragments transfected into BHK cells as described previously (52). A second incubation was done at 37°C for 60 min in 12-well plates, followed by an agarose-DMEM (containing 2% fetal calf serum and 0.6% final agarose concentration) overlay. Cells were incubated for 4 days before formaldehyde fixation, staining with crystal violet, and plaque counting. Serum titers reducing ZIKV PFU by ≥50% compared to controls in any dilution were considered positive. NS1 ELISA ratios of sera tested by PRNT did not differ significantly from those not tested by PRNT (*P* = 0.20 by *t* test).

10.1128/mBio.01390-17.2 FIG S2  ELISA ratios. The numbers of sera tested for ZIKV antibodies (NS1 antigen) and CHIKV antibodies were as follows: 96 in 2013, 89 in 2014, 92 in 2015, and 263 in 2016 for HIV-infected patients (HIV); 257 for non-microcephaly pregnancies (PRG); 19 for microcephaly pregnancies (MC); 55 for tuberculosis patients (TBC); and 39 for university employees (UNI). The numbers of sera tested for DENV were 84 in 2013, 82 in 2014, 69 in 2015, and 110 in 2016 for HIV; 69 for PRG, 1 for MC, 8 for TBC, and 18 for UNI. Dashed lines indicate signal-to-cutoff ratios of ≥1.1, which are considered positive, and ratios between 0.8 and 1.1, which are considered borderline by the manufacturer (conservatively considered negative in this study together with all ratios of <0.8). Horizontal lines in plots indicate mean ratios. Only clearly ZIKV-negative specimens yielding signal-to-cutoff ratios of <0.8 as stated in the manufacturer’s instructions were used for assessments of DENV seroprevalence. Download FIG S2, PDF file, 1.4 MB. Copyright © 2017 Martins Netto et al. 2017 Martins Netto et al. This content is distributed under the terms of the Creative Commons Attribution 4.0 International license.

### Georeference and demographic data.

The home addresses of study participants were georeferenced onto spatial units (human development units [HDUs]) according to census data from the Instituto Brasileiro de Geografia e Estatística, dividing the metropolitan region of Salvador, Brazil, into socioeconomically homogenous areas, taking into account a minimum of 400 permanent households at a first classification step, and socioeconomic homogeneity at a second step, provided within the Brazilian Human development atlas (http://www.atlasbrasil.org.br/2013/pt/consulta/) from the United Nations Development Programme (UNDP). HDUs are described by seven different categories: population, demography, housing, labor, education, income, and vulnerability. Maps were generated using ArcGIS 10.3 (ESRI, Redlands, CA, USA).

### Statistical analyses.

Statistical analyses included χ^2^ and Fisher’s exact tests for comparisons of seroprevalence rates (EpiInfo V7.2; http://www.cdc.gov/epiinfo), two-tailed Mann-Whitney U tests for comparisons of sociodemographic indicators and logistic regression with stepwise backward elimination of variables for multivariate analyses (SPSS V23; IBM, Ehningen, Germany), done on one variable per HDU category, selected according to highest *P* values in bivariate comparisons. Diagnostic test parameters were calculated using OpenEpi (http://www.openepi.com).

### Transmission dynamic modeling.

ZIKV outbreak dynamics were analyzed using a susceptible-exposed-infectious-recovered (SEIR) model. The vector population was not explicitly taken into account, but variation in mosquito numbers was modeled through annual seasonal forces acting on the transmission rate. The model was implemented in a Bayesian framework using the LibBi library via the RBi and RBi.helpers packages (53–55). The model was jointly fitted to reported ZIKV incidence data from Salvador, Brazil, from the beginning of January 2015 to the end of October 2015 and the proportion of individuals (401/633) who were seropositive to ZIKV in 2016. Incidence was fitted using a Poisson likelihood with overdispersion and approximated with a truncated Gaussian distribution, and seroprevalence was fitted using a binomial likelihood. Informative prior probability distributions were used for the delay between infection and infectiousness (the sum of the mosquito-to-human generation time and the intrinsic incubation period) centered around 17.8 days, and the infectious period in humans centered around 4.7 days, respectively (8), and for the peak of seasonality in mid-May based on dengue transmission dynamics in Salvador, Brazil. Uniform prior probability distributions were used for the amplitude of seasonality, proportion of cases reported, and basic reproduction number, *R*_0_. Regularizing prior probability distributions were used for the initial numbers of infected and overdispersion of reporting. All model parameters were estimated using Markov chain Monte Carlo. Full prior and posterior probability distributions are shown in Fig. S3.

10.1128/mBio.01390-17.3 FIG S3  Parameter estimates for the fitted mathematical model. Posterior and prior parameter probability density plots for the model. D_inc_, duration between infection caused by mosquitoes in a human and the subsequent human case to become infectious; D_inf_, duration of human infectiousness; R_0_, basic reproduction number; r, proportion of cases reported; ϕ, overdispersion of reporting; Peak week, week of peak Zika transmission; Peak amplitude, peak amplitude (as a proportion of the average transmission rate); I_0_, initial number of infectives. Download FIG S3, PDF file, 0.04 MB. Copyright © 2017 Martins Netto et al. 2017 Martins Netto et al. This content is distributed under the terms of the Creative Commons Attribution 4.0 International license.

### Data availability.

A document containing the code necessary to reproduce the modeling results is provided in Data Set S1.

10.1128/mBio.01390-17.8 Data Set S1  Script to reproduce mathematical modeling in R. Download Data Set S1, PDF file, 0.6 MB. Copyright © 2017 Martins Netto et al. 2017 Martins Netto et al. This content is distributed under the terms of the Creative Commons Attribution 4.0 International license.
