# Supplementary material for: High Zika Virus Seroprevalence in Salvador, Northeastern Brazil Limits the Potential for Further Outbreaks
Source: mBio. 2017 Nov 14;8(6):e01390-17. doi: 10.1128/mBio.01390-17 (PMC5686533; doi:10.1128/mBio.01390-17)
Supplement: FIG S1 [file mbo006173587sf1.pdf]

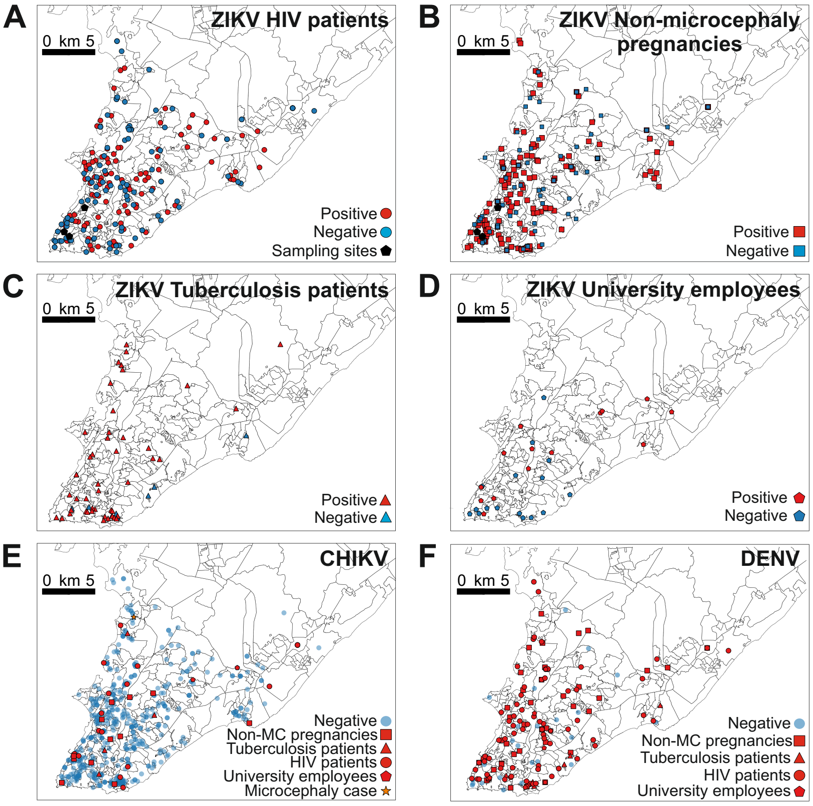

Supplementary Figure 1. Spatial distribution of samples per subpopulation in Salvador, Bahia

Panels A-D show origin of samples for all subpopulations, positive (red), negative (blue). Panels E and F show all positive samples in red and different shapes, whereas negative samples of all subpopulations are shown in shades of blue for clarity of presentation. Orange star in panel E shows the single CHIKV-positive microcephaly pregnancy. DENV includes only ZIKV-negative specimens due to cross-reactivity of the DENV ELISA with ZIKV antibodies. HIV-infected patients from 2013-2015 are not shown due to low ZIKV positivity.
