# Supplementary material for: High Zika Virus Seroprevalence in Salvador, Northeastern Brazil Limits the Potential for Further Outbreaks
Source: mBio. 2017 Nov 14;8(6):e01390-17. doi: 10.1128/mBio.01390-17 (PMC5686533; doi:10.1128/mBio.01390-17)
Supplement: TABLE S1 [file mbo006173587st1.docx]

**Supplementary Table 1. Zika virus test results in HIV-infected patients**

| Subpopulation | | Median age [years] (IQR*) | ZIKV NS1 IgG ELISA | | Competitive ZIKV E  ELISA | | ZIKV PRNT | |
| --- | --- | --- | --- | --- | --- | --- | --- | --- |
|  | |  | **n/total** | **%** | **n/total** | **%** | **n/total** | **%** |
|  | HIV patients 2013 | 36.7 (16.4) | 7/96 | (7.3) | 4 /96 | (4.2) | - | - |
|  | HIV patients 2014 | 38.8 (17.8) | 2/89 | (2.3) | 4/89 | (4.5) | - | - |
|  | HIV patients 2015 | 36.6 (17.4) | 16/92 | (17.4) | 16/92 | (17.4) | - | - |
|  | **Total retrospective** |  | 277 |  | 277 |  |  |  |
|  | HIV patients 2016 | 44.7 (15.4) | 139/263 | (52.9) | 113/263 | (43.0) | 31/61 | (50.8) |
|  | **Total** |  | 540 |  | 540 |  | 61 |  |
