# Supplementary material for: High Zika Virus Seroprevalence in Salvador, Northeastern Brazil Limits the Potential for Further Outbreaks
Source: mBio. 2017 Nov 14;8(6):e01390-17. doi: 10.1128/mBio.01390-17 (PMC5686533; doi:10.1128/mBio.01390-17)
Supplement: TABLE S2 [file mbo006173587st2.docx]

**Supplementary Table 2. Gender distribution in prospectively sampled subpopulations**

|  |  | Female | | Male | |  |
| --- | --- | --- | --- | --- | --- | --- |
| Subpopulation | **N** | **n (%)** | **ZIKV positive n (%)** | **n (%)** | **ZIKV positive n (%)** | **Sig 2-tailed** |
| MC pregnancies | 19 | 17 (89.5) | 16 (94.1) | 2 (10.5) | 2 (100) | n/a |
| Non-MC pregnancies | 257 | 255 (99.2) | 177 (69.4) | 2 (0.8) | 1 (50.0) | n/a |
| HIV patients | 263 | 108 (41.1) | 51 (47.2) | 155 (58.9) | 88 (56.8) | 0.16 |
| Tuberculosis patients | 55 | 24 (43.6) | 19 (79.2) | 31 (56.4) | 28 (90.3) | 0.27 |
| University employees | 39 | 33 (84.6) | 18 (54.5) | 6 (15.4) | 1 (16.7) | 0.18 |
| Total | 633 | 437 (69.0) |  | 196 (31.0) |  | 0.51 |

MC, microcephaly; Sig, significance; Data based on NS1-antigen ELISA
