# Supplementary material for: High Zika Virus Seroprevalence in Salvador, Northeastern Brazil Limits the Potential for Further Outbreaks
Source: mBio. 2017 Nov 14;8(6):e01390-17. doi: 10.1128/mBio.01390-17 (PMC5686533; doi:10.1128/mBio.01390-17)
Supplement: TABLE S3 [file mbo006173587st3.docx]

**Supplementary Table 3. Age distribution of study participants compared to the population of Salvador**

| Age strata | Salvador population* | This study | ZIKV IgM | ZIKV IgG | CHIKV IgG | DENV total^#^ | DENV IgG |
| --- | --- | --- | --- | --- | --- | --- | --- |
|  | **N (%)** | **n (%)** | **n (%)** | **n (%)** | **n (%)** | **n** | **n (%)** |
| 0-9 | 343,882 (12.9) | 6 (1.0) | 1 (16.6) | 5 (83.3) | 4 (66.6) | 1 | 0 (0) |
| 10-19 | 425,477 (16.0) | 33 (5.3) | 1 (3) | 18 (54.5) | 1 (3) | 11 | 9 (81.8) |
| 20-29 | 537,342 (20.2) | 155 (24.8) | 0 (0) | 103 (66.5) | 9 (5.8) | 46 | 33 (71.7) |
| 30-39 | 480,679 (18.1) | 193 (30.9) | 2 (1) | 126 (65.3) | 12 (6.2) | 63 | 53 (84.1) |
| 40-49 | 375,101 (14.1) | 120 (19.2) | 2 (1.7) | 69 (57.5) | 7 (5.9) | 48 | 31 (64.6) |
| 50-59 | 265,529 (10.0) | 84 (13.4) | 2 (2.4) | 55 (65.5) | 7 (8.3) | 23 | 18 (78.3) |
| 60-84 | 231,729 (8.7) | 34 (5.4) | 0 (0) | 19 (55.9) | 6 (17.6) | 15 | 12 (80.0) |
| Total | 2,659,739 | 625^*^ | 8 (1.3) | 395 (63.2) | 47 (7.4) | 206 | 156 (75.7) |

*Census information taken from <http://www.ibge.gov.br/estadosat/temas.php?sigla=ba&tema=sinopse_censodemog2010>; Because the majority of study participants originated from Salvador, ages were compared to Salvador, not to all of the state of Bahia

#including only ZIKV-negative specimens due to cross-reactivity of the DENV ELISA with ZIKV antibodies

^*^age information was missing for eight participants

All serological data based on Euroimmun ELISAs
