# Supplementary material for: High Zika Virus Seroprevalence in Salvador, Northeastern Brazil Limits the Potential for Further Outbreaks
Source: mBio. 2017 Nov 14;8(6):e01390-17. doi: 10.1128/mBio.01390-17 (PMC5686533; doi:10.1128/mBio.01390-17)
Supplement: TABLE S4 [file mbo006173587st4.docx]

**Supplementary Table 4. Association between ZIKV infections and socio-demographic indicators**

| **Category** | **Indicator** | **Median negative HDUs** | **Median positive HDUs** | **Mann-Whitney U** | **p** |
| --- | --- | --- | --- | --- | --- |
| Demography | Dependency ratio | 35.38 | 37.15 | 1227.50 | .193 |
|  | Child mortality | 15.43 | 17.79 | 1333.50 | .455 |
|  | Life expectancy at birth | 76.44 | 75.02 | 1333.50 | .455 |
|  | Probability of survival up to 60 years | 88.32 | 87.46 | 1335.50 | .461 |
|  | Probability of survival up to 40 years | 96.06 | 95.77 | 1336.50 | .465 |
|  | Infant mortality | 13.25 | 15.30 | 1343.50 | .487 |
|  | Total fertility rate | 1.34 | 1.45 | 1376.50 | .602 |
|  | Aging rate | 5.77 | 5.95 | 1400.50 | .692 |
| Education | net basic education attendance frequency | 90.76 | 87.38 | 1003.50 | .013 |
|  | gross secondary education attendance frequency | 84.98 | 78.89 | 1085.50 | .041 |
|  | % of 11-13 year-olds having completed primary education or attending its final years | 89.25 | 84.85 | 1098.50 | .048 |
|  | % of 11- 13 year-olds at the end of primary education | 91.41 | 88.62 | 1134.50 | .073 |
|  | net secondary education attendance frequency | 44.24 | 35.62 | 1137.00 | .076 |
|  | % of 15-17 year-olds attending school | 90.05 | 87.97 | 1145.00 | .083 |
|  | Illiteracy rate - 18 to 24 years old | 0.81 | 1.18 | 1173.00 | .112 |
|  | Net secondary education rate | 53.74 | 47.59 | 1203.50 | .153 |
|  | % of 6-14 year-olds attending school | 96.10 | 94.60 | 1210.00 | .163 |
|  | Illiteracy rate - aged 15 years or older | 2.45 | 3.83 | 1211.50 | .166 |
|  | Illiteracy rate - aged 18 years or older | 2.55 | 3.99 | 1212.50 | .167 |
|  | Illiteracy rate - aged 25 years or older | 2.92 | 4.52 | 1219.50 | .179 |
|  | % of mothers that are head of households. without completing primary education and having children under 15 years of age | 23.57 | 27.93 | 1225.50 | .189 |
|  | Illiteracy rate - 25 to 29 years old | 1.09 | 1.40 | 1238.50 | .213 |
|  | net primary education attendance frequency | 93.47 | 91.80 | 1239.50 | .215 |
|  | Expected years of schooling | 10.06 | 9.65 | 1249.00 | .234 |
|  | % of 15-17 year-olds having completed primary education | 55.90 | 49.35 | 1251.50 | .239 |
|  | % of mothers that are head of households. without completing primary education and having children under 15 years of age | 9.06 | 11.78 | 1267.50 | .274 |
|  | % of 6-17 year-olds attending basic education with 2 year delay | 19.86 | 23.52 | 1281.00 | .307 |
|  | % of 18-20 year-olds having completed secondary education | 46.86 | 39.15 | 1284.50 | .316 |
|  | Net basic education rate | 96.24 | 95.26 | 1286.00 | .319 |
|  | Gross primary education rate | 120.53 | 126.43 | 1298.00 | .351 |
|  | % of 25 year-olds or older having completed higher education | 9.61 | 6.03 | 1302.50 | .363 |
|  | Illiteracy rate - 15 to 17 years old | 1.35 | 1.41 | 1306.00 | .373 |
|  | gross higher education attendance frequency | 40.77 | 25.99 | 1310.50 | .386 |
|  | % of 18-24 year-olds having completed secondary education | 60.86 | 52.06 | 1313.50 | .394 |
|  | net higher education attendance frequency | 17.85 | 8.00 | 1314.50 | .397 |
|  | % of 18-24 year-olds attending school | 37.88 | 35.04 | 1314.50 | .397 |
|  | % of 18-24 year-olds attending secondary school | 9.60 | 8.56 | 1319.50 | .412 |
|  | % of 6-14 year-olds attending primary school with delay of 2 years or more | 13.67 | 18.00 | 1325.50 | .430 |
|  | Gross basic education attendance frequency | 108.63 | 108.36 | 1326.50 | .433 |
|  | % of 18-24 year-olds having completed primary education | 83.07 | 79.85 | 1327.50 | .436 |
|  | % of people 18 years or older without basic education | 17.85 | 22.74 | 1337.00 | .466 |
|  | % of children aged 0-5 years not attending school | 50.32 | 49.44 | 1337.50 | .468 |
|  | % of 0-5 year-olds attending school | 49.68 | 50.56 | 1337.50 | .468 |
|  | Net primary education rate | 97.82 | 97.45 | 1355.00 | .526 |
|  | % of people 15-24 years of age who neither study nor work and are vulnerable to poverty | 7.82 | 10.73 | 1356.50 | .531 |
|  | % of 18 year-olds or older having completed primary education | 75.86 | 68.36 | 1358.50 | .538 |
|  | % of employed with secondary education completed - aged 18 years or older | 55.61 | 48.55 | 1362.50 | .552 |
|  | Gross basic education rate | 120.66 | 123.04 | 1363.50 | .555 |
|  | % of 25 year-olds or older having completed primary education | 73.28 | 66.67 | 1364.50 | .559 |
|  | gross primary education attendance frequency | 116.51 | 119.57 | 1367.50 | .570 |
|  | % of 25 year-olds or older having completed secondary education | 54.13 | 48.12 | 1379.50 | .613 |
|  | % of people in households where no one has completed primary education | 9.80 | 12.93 | 1385.00 | .633 |
|  | Population 11-14 years old | 2.05 | 2.02 | 1387.00 | .641 |
|  | Gross secondary education rate | 115.99 | 112.98 | 1389.50 | .650 |
|  | % of children in households where no one has completed primary education | 11.14 | 16.38 | 1392.50 | .662 |
|  | % of 15-17 year-olds attending primary school | 32.08 | 33.83 | 1406.50 | .716 |
|  | % of 18-24 year-olds attending primary school | 3.93 | 4.39 | 1423.00 | .781 |
|  | % of 5-6 year-olds attending school | 95.53 | 95.15 | 1448.00 | .883 |
|  | % of 25-29 year-olds attending school | 17.17 | 16.78 | 1455.00 | .912 |
|  | % of children aged 6-14 years not attending school | 3.21 | 2.96 | 1473.50 | .990 |
|  | % of 6-14 year-olds attending school | 96.80 | 97.04 | 1473.50 | .990 |
|  | Population 15- 24 that do not study or work | 31.89 | 31.53 | 1474.50 | .994 |
| Housing | % of the population in households with garbage collection | 98.54 | 97.47 | 1103.50 | .050 |
|  | % of people in households with inadequate water supply and sanitation | 0.00 | 0.10 | 1121.50 | .051 |
|  | % of the population in households with piped water and toilet | 97.51 | 95.88 | 1132.50 | .072 |
|  | % of the population in households with piped water | 99.81 | 99.65 | 1167.50 | .106 |
|  | % of people in households vulnerable to poverty and dependent on an elderly member of the family | 1.23 | 1.74 | 1185.00 | .127 |
|  | % of people in households vulnerable to poverty and dependent on an elderly member of the family | 69.67 | 60.59 | 1277.00 | .297 |
|  | % of households with population density> 2 | 24.64 | 28.84 | 1277.50 | .298 |
|  | % of the population in households with electricity | 99.92 | 99.95 | 1311.50 | .366 |
|  | % of the population in households without electricity | 0.08 | 0.05 | 1311.50 | .366 |
|  | % of people in households vulnerable to poverty and where no one has completed primary education | 4.22 | 6.45 | 1391.50 | .658 |
|  | % of the population in households with inadequate walls | 0.40 | 0.35 | 1429.50 | .807 |
| Income | The richest 10% people share of total income. | 34.70 | 33.81 | 1076.50 | .036 |
|  | The poorest 80% people's share of total income | 49.14 | 49.83 | 1128.50 | .069 |
|  | The richest 20% people's share of total income | 50.86 | 50.17 | 1128.50 | .069 |
|  | % of employed with no income - aged 18 years or older | 0.96 | 1.27 | 1148.00 | .086 |
|  | % of income that is labour income | 78.78 | 76.56 | 1185.50 | .128 |
|  | The poorest 60% people's share of total income | 27.20 | 27.86 | 1202.00 | .151 |
|  | % of employed with income of up to 5 minimum wages - aged 18 years or older | 92.87 | 96.42 | 1225.00 | .188 |
|  | Index Theil-L of labour income - aged 18 years or older | 0.33 | 0.29 | 1227.00 | .191 |
|  | Ratio richest 10% of people / poorest 40% of people | 10.19 | 10.01 | 1243.00 | .222 |
|  | Ratio richest 20% of people / poorest 40% of people | 7.59 | 7.51 | 1258.00 | .253 |
|  | % of employed with income of up to two minimum wages - 18 years of age or older | 66.13 | 75.47 | 1278.00 | .299 |
|  | % of employed with income up to 3 minimum wages - aged 18 years or older | 82.37 | 88.77 | 1283.50 | .313 |
|  | Average income of the employed - 18 years of age or older | 1147.52 | 879.34 | 1284.50 | .316 |
|  | Mean per capita income of the poorest quintile of the population | 155.26 | 131.40 | 1299.50 | .355 |
|  | Mean per capita income of the top income quintile | 1878.62 | 1437.14 | 1307.50 | .377 |
|  | Top per capita income of the top income quintile | 2648.52 | 1971.65 | 1307.50 | .377 |
|  | Per capita income | 715.67 | 561.45 | 1324.50 | .427 |
|  | Top per capita income of the poorest quintile of the population | 229.50 | 200.00 | 1339.00 | .472 |
|  | Lowest per capita income of the top income decile | 1419.42 | 1070.00 | 1342.50 | .484 |
|  | Per capita income of the extremely poor people | 36.36 | 39.18 | 1253.00 | .485 |
|  | The poorest 20% people's share of total income | 4.53 | 4.20 | 1349.50 | .507 |
|  | Top per capita income of the 3rd income quintile | 491.46 | 398.71 | 1353.50 | .521 |
|  | Mean per capita income of the 2nd income quintile | 307.55 | 254.42 | 1357.50 | .535 |
|  | The poorest 40% people's share of total income | 13.36 | 13.33 | 1362.50 | .552 |
|  | Maximum per capita income of the poorest 3rd fifth | 596.67 | 500.00 | 1363.00 | .554 |
|  | Top per capita income of the 2nd income quintile | 388.34 | 324.00 | 1364.00 | .557 |
|  | Top per capita income of the 4th income quintile | 987.50 | 750.00 | 1372.00 | .586 |
|  | Mean per capita income of the 4th income quintile | 772.21 | 603.06 | 1375.50 | .598 |
| Labour | Economically-active population 10-14 years old | 22.00 | 44.00 | 884.50 | .002 |
|  | Economically-active population aged 18 years or older | 3807.00 | 6693.00 | 945.00 | .005 |
|  | Activity Rate- age 10 to 14 years | 4.15 | 5.14 | 1022.50 | .017 |
|  | Economically-active population 15-17 years old | 52.50 | 114.00 | 1078.50 | .037 |
|  | % of public sector workers - 18 years of age or older | 5.52 | 3.55 | 1142.00 | .080 |
|  | Degree of formal employment - aged 18 years or older | 69.19 | 66.28 | 1172.50 | .112 |
|  | Activity Rate- age 18 or older | 70.86 | 70.32 | 1179.50 | .120 |
|  | % self-employed people - aged 18 years or older | 18.54 | 19.24 | 1183.50 | .125 |
|  | Gini index | 0.46 | 0.45 | 1210.00 | .161 |
|  | % of employed in SIUP - aged 18 years or older | 1.06 | 1.06 | 1244.50 | .225 |
|  | Unemployment Rate - 10 to 14 years | 29.23 | 34.58 | 968.50 | .248 |
|  | % of people that spend more than an hour to go to work | 16.17 | 19.32 | 1270.00 | .280 |
|  | % of employed with higher education degree - aged 18 years or older | 11.51 | 7.16 | 1281.50 | .308 |
|  | Degree of formal employment – population aged 18 years or older | 57.39 | 56.88 | 1293.50 | .339 |
|  | % of employed in the manufacturing industry - aged 18 years or older | 7.00 | 6.42 | 1300.00 | .356 |
|  | Degree of informal employment – population aged 18 years or older | 14.90 | 16.90 | 1312.00 | .390 |
|  | Theil index-L | 0.39 | 0.39 | 1313.50 | .393 |
|  | % of employed earning up to 1 minimum wage - aged 18 years or older | 12.87 | 16.97 | 1317.50 | .406 |
|  | Unemployment Rate - 18 to 24 | 24.32 | 26.61 | 1329.50 | .443 |
|  | % of employed in the mineral extraction sector - aged 18 years or older | 0.42 | 0.46 | 1333.50 | .455 |
|  | % of employed in the construction sector - aged 18 years or older | 6.23 | 8.04 | 1335.00 | .460 |
|  | Activity Rate- age 25 to 29 | 83.07 | 82.19 | 1342.00 | .482 |
|  | Activity Rate- age 18 to 24 | 68.97 | 68.60 | 1353.50 | .521 |
|  | % of employed with primary education completed - aged 18 years or older | 81.47 | 73.98 | 1370.50 | .580 |
|  | % of employed with secondary education completed - aged 18 years or older | 62.10 | 55.30 | 1370.50 | .580 |
|  | % of employed in the service sector - aged 18 years or older | 57.17 | 56.22 | 1372.50 | .587 |
|  | Activity Rate- age 15 to 17 | 23.77 | 22.74 | 1394.50 | .669 |
|  | Unemployment rate - aged 18 years or older | 12.40 | 12.85 | 1400.00 | .690 |
|  | Unemployment Rate - 15 to 17 years | 44.87 | 43.34 | 1407.50 | .720 |
|  | Unemployment Rate - 25 to 29 years | 16.03 | 15.83 | 1411.50 | .735 |
|  | % of employed in the trade sector - aged 18 years or older | 16.95 | 17.38 | 1430.50 | .812 |
|  | % employers - aged 18 years or older | 0.98 | 0.87 | 1444.50 | .869 |
|  | % employed in the agricultural sector - aged 18 years or older | 0.45 | 0.51 | 1446.00 | .875 |
| MHDI | EHDI Education municipal human development index | 0.73 | 0.67 | 1275.50 | .293 |
|  | MHDI Municipal human development index | 0.76 | 0.73 | 1304.50 | .369 |
|  | IHDI Income municipal human development index | 0.72 | 0.68 | 1323.00 | .423 |
|  | LHDI Longevity municipal human development index | 0.86 | 0.83 | 1334.50 | .458 |
| Population | Urban population | 6351.50 | 13853.00 | 916.00 | .003 |
|  | Female population 20-24 years old | 338.50 | 674.00 | 914.00 | .003 |
|  | Female population 45-49 years old | 258.00 | 461.00 | 923.00 | .004 |
|  | Population 18-24 years old | 911.00 | 1733.00 | 923.00 | .004 |
|  | Female population 50-54 years old | 237.00 | 389.00 | 924.50 | .004 |
|  | Female resident population | 3676.00 | 7184.00 | 928.00 | .004 |
|  | Women aged 15 years or older | 3047.50 | 5580.00 | 930.00 | .004 |
|  | Female population 55-59 years old | 175.00 | 323.00 | 934.00 | .005 |
|  | Women aged 25 years or older | 2568.00 | 4456.00 | 938.00 | .005 |
|  | Male population 20-24 years old | 320.50 | 597.00 | 938.50 | .005 |
|  | Male population 25-29 years old | 371.50 | 681.00 | 943.50 | .005 |
|  | Male population 45-49 years old | 217.00 | 409.00 | 944.00 | .005 |
|  | Male population 55-59 years old | 151.50 | 255.00 | 944.00 | .005 |
|  | Population aged 10 years or older | 6154.00 | 11540.00 | 944.00 | .005 |
|  | Total population | 7081.00 | 13853.00 | 947.00 | .006 |
|  | Population aged 18 years or older | 5237.50 | 9779.00 | 949.00 | .006 |
|  | Population aged 15 years or older | 5585.50 | 10558.00 | 950.00 | .006 |
|  | Female population 15-19 years old | 256.00 | 535.00 | 952.00 | .006 |
|  | Female population 40-44 years old | 289.00 | 531.00 | 952.50 | .006 |
|  | Population aged 25 years or older | 4459.50 | 8001.00 | 953.00 | .006 |
|  | Male resident population | 3405.00 | 6635.00 | 954.00 | .006 |
|  | Male population 60-64 years old | 98.50 | 182.00 | 954.50 | .006 |
|  | Male population 15-19 years old | 271.50 | 520.00 | 955.00 | .006 |
|  | Male population 50-54 years old | 176.50 | 330.00 | 956.00 | .006 |
|  | Female population 35-39 years old | 294.50 | 558.00 | 960.00 | .007 |
|  | Male population 40-44 years old | 234.00 | 433.00 | 964.00 | .007 |
|  | Female population 25-29 years old | 418.50 | 736.00 | 964.50 | .007 |
|  | Female population 60-64 years old | 116.00 | 230.00 | 965.50 | .007 |
|  | Population 15-17 years old | 307.50 | 649.00 | 969.50 | .008 |
|  | Male population 35-39 years old | 251.00 | 464.00 | 969.50 | .008 |
|  | Female population 75-79 years old | 47.50 | 97.00 | 979.00 | .009 |
|  | Female population 5-9 years old | 199.00 | 405.00 | 982.50 | .010 |
|  | Population aged 65 years or older | 432.50 | 884.00 | 984.00 | .010 |
|  | Female population aged 80 years and older | 61.00 | 122.00 | 986.00 | .010 |
|  | Female population 65-69 years old | 89.00 | 173.00 | 986.50 | .010 |
|  | Population under 1 year of age | 79.00 | 149.00 | 987.50 | .010 |
|  | Female population 70-74 years old | 67.50 | 130.00 | 988.00 | .011 |
|  | Population 6 years of age | 79.50 | 170.00 | 988.50 | .011 |
|  | Female population 30-34 years old | 400.50 | 691.00 | 991.50 | .011 |
|  | Population 6-17 years of age | 1109.00 | 2322.00 | 991.50 | .011 |
|  | Female population 10-14 years old | 227.50 | 508.00 | 993.00 | .011 |
|  | Male population 70-74 years old | 57.50 | 84.00 | 996.00 | .012 |
|  | Male population 0-4 years old | 196.50 | 408.00 | 997.00 | .012 |
|  | Population 5 years of age | 77.00 | 158.00 | 998.50 | .012 |
|  | Population 6-10 years old | 428.00 | 893.00 | 998.50 | .012 |
|  | Population 11-14 years old | 373.50 | 802.00 | 999.50 | .013 |
|  | Male population 10-14 years old | 238.50 | 506.00 | 1005.00 | .014 |
|  | Male population 5-9 years old | 211.50 | 462.00 | 1006.00 | .014 |
|  | Population 1-3 years old | 223.50 | 459.00 | 1006.00 | .014 |
|  | Female population 0-4 years old | 191.00 | 370.00 | 1007.50 | .014 |
|  | Male population 75-79 years old | 28.50 | 54.00 | 1009.00 | .014 |
|  | Male population 65-69 years old | 63.00 | 118.00 | 1009.50 | .014 |
|  | Male population 30-34 years old | 360.50 | 595.00 | 1009.50 | .014 |
|  | Male population aged 80 years and older | 31.00 | 53.00 | 1016.00 | .016 |
|  | Population 4 years of age | 82.50 | 159.00 | 1032.00 | .020 |
|  | % of women aged 10-17 years having children | 0.31 | 1.23 | 1083.50 | .038 |
| Vulnerability | % of people vulnerable to poverty that spend more than an hour to go to work | 0.85 | 1.87 | 1205.50 | .156 |
|  | % of extremely poor children | 4.38 | 5.55 | 960.50 | .158 |
|  | % of poor children | 10.83 | 17.61 | 1215.00 | .189 |
|  | % of poor people | 6.64 | 9.93 | 1217.00 | .192 |
|  | % of extremely poor people | 2.10 | 2.85 | 1222.00 | .385 |
|  | % of children vulnerable to poverty | 39.37 | 45.62 | 1331.50 | .449 |
|  | % of people vulnerable to poverty | 25.39 | 32.30 | 1339.50 | .474 |
|  |  |  |  |  |  |

The number of compared HDUs was generally 24 for ZIKV-negative HDUs and 123 for ZIKV-positive HDUs. Serological data are based on Euroimmun ZIKV IgM and IgG ELISAs.
