# Supplementary material for: High Zika Virus Seroprevalence in Salvador, Northeastern Brazil Limits the Potential for Further Outbreaks
Source: mBio. 2017 Nov 14;8(6):e01390-17. doi: 10.1128/mBio.01390-17 (PMC5686533; doi:10.1128/mBio.01390-17)
Supplement: FIG S2 [file mbo006173587sf2.pdf]

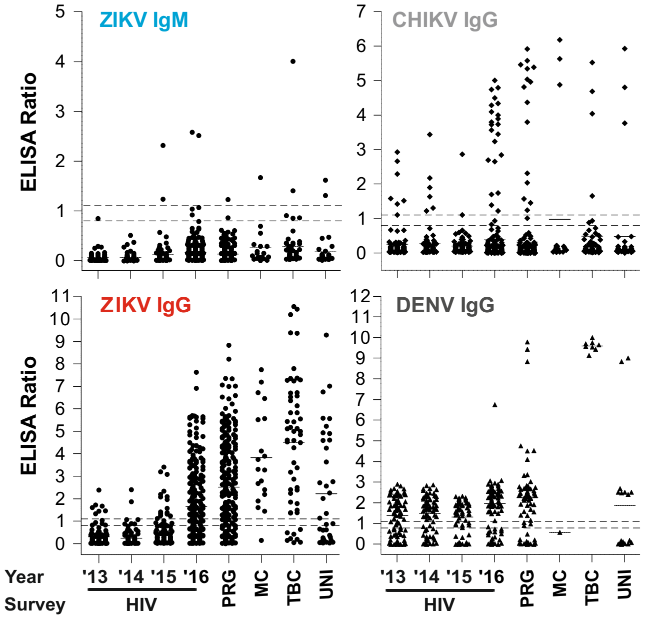

Supplementary Figure 2. ELISA ratios

Numbers of sera tested for ZIKV (NS1 antigen) and CHIKV were: HIV-infected patients (HIV) 2013=96, 2014=89, 2015=92, 2016=263, non-microcephaly pregnancies (PRG)=257, microcephaly pregnancies (MC)=19, Tuberculosis patients (TBC)=55 and university employees (UNI)=39. Numbers of sera tested for DENV were: HIV 2013=84, 2014=82, 2015=69, 2016=110, PRG=69, MC=1, TBC=8 and UNI=18. Dashed lines indicate signal-to-cut-off ratios of =1.1 considered positive, and ratios between 0.8-1.1 considered borderline by the manufacturer (considered negative in this study together with all ratios <0.8). Horizontal lines in plots indicate mean ratios. Only clearly ZIKV-negative specimens yielding signal-to-cut-off ratios <0.8 as stated in the manufacturer's instructions were used for assessments of DENV seroprevalence.
