# Supplementary material for: High Zika Virus Seroprevalence in Salvador, Northeastern Brazil Limits the Potential for Further Outbreaks
Source: mBio. 2017 Nov 14;8(6):e01390-17. doi: 10.1128/mBio.01390-17 (PMC5686533; doi:10.1128/mBio.01390-17)
Supplement: FIG S3 [file mbo006173587sf3.pdf]

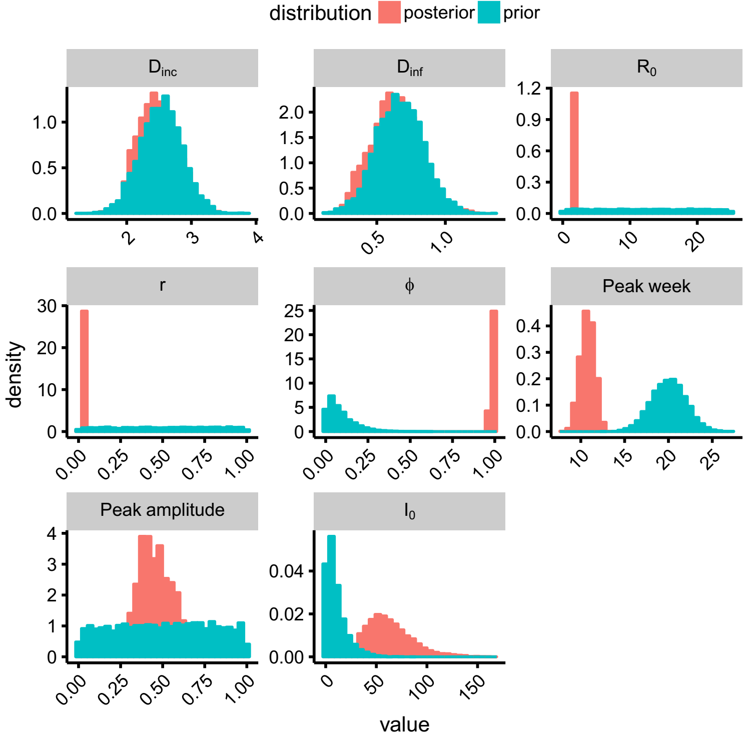

Supplementary Figure 3. Parameter estimates for the fitted mathematical model

Posterior and prior parameter probability density plots for the model. D_{inc}: duration between mosquito infection caused by a human and subsequent human case to become infectious. D_{inf}: duration of human infectiousness. R_0: basic reproduction number. r: proportion of cases reported. ?: overdispersion of reporting. Peak week: Week of peak Zika transmission. Peak amplitude: Peak amplitude (as proportion of average transmission rate), I_0: initial number of infectives.
