# Supplementary material for: High Zika Virus Seroprevalence in Salvador, Northeastern Brazil Limits the Potential for Further Outbreaks
Source: mBio. 2017 Nov 14;8(6):e01390-17. doi: 10.1128/mBio.01390-17 (PMC5686533; doi:10.1128/mBio.01390-17)
Supplement: Data Set S1 [file mbo006173587sd1.pdf]

Supplementary material: Modelling results

High seroprevalence against Zika virus in Salvador, north-eastern Brazil limits the potential for further outbreaks

Eduardo Martins Netto, Andres Moreira-Soto, Celia Pedroso, Christoph Höser, Sebastian Funk, Adam J. Kucharski, Alexandra Rockstroh, Beate M. Kümmerer, Gilmara Souza Sampaio, Estela Luz, Sara Nunes Vaz, Juarez Pereira Dias, Fernanda Anjos Bastos, Renata Cabral, Thomas Kistemann, Sebastian Ulbert, Xavier de Lamballerie, Thomas Jaenisch, Oliver J. Brady, Christian Drosten, Manoel Sarno, Carlos Brites, Jan Felix Drexler

- Introduction
    - Required packages
- Model fitting
    - Generate posterior samples
- Prediction
- \(R_0\) calculations
- Plots
    - Predictions
    - Parameter plots
- Session information

Introduction

This document contains the R code necessary to reproduce the modelling results in "High seroprevalence against Zika virus in Salvador, north-eastern Brazil limits the potential for further outbreaks".

PRO version Are you a developer? Try out the HTML to PDF API pdfcrowd.com

Required packages

Install required packages

```
cran_packages <- c("magrittr", "cowplot", "scales", "dplyr")
github_packages <- c("sbfnk/RBi.helpers")
```

```
for (package in cran_packages) {
    install.packages(package)
}

library("devtools")

for (package in github_packages) {
    install_github(package)
}
```

Load required packages

```
for (package in c(cran_packages, github_packages)) {
    library(tolower(sub("^.*/", "", package)), character.only = TRUE)
}
```

Model fitting

Generate posterior samples

PRO version Are you a developer? Try out the HTML to PDF API pdfcrowd.com

Set the number of samples in the serological study

```
n_serology = 633
```

Read observations and model

```
model_dir <- path.expand("~/code/vbd/bi")
obs <- readRDS("fit_data.rds")
vbd_model <- bi_model(paste(model_dir, "vbd.bi", sep = "/"))
```

Set population size, initial population immune, and proportion of the population at risk to constants

```
vbd_model %<>% fix(N = 2675000, p_p_immune = 0.06, p_p_risk = 1)
```

Print data

```
obs
```

```
## $Incidence
## # A tibble: 43 x 2
##      time value
##     <int> <dbl>
##  1      1     9
##  2      2     6
##  3      3     8
##  4      4     9
##  5      5    18
##  6      6    17
##  7      7     7
##  8      8    15
```

PRO version Are you a developer? Try out the HTML to PDF API pdfcrowd.com

```
## 9      9    14
## 10    10    10
## 11    11    43
## 12    12   107
## 13    13   147
## 14    14   161
## 15    15   298
## 16    16   440
## 17    17   408
## 18    18   706
## 19    19   777
## 20    20  1020
## 21    21  1033
## 22    22  1143
## 23    23  1100
## 24    24  1272
## 25    25  1265
## 26    26  1595
## 27    27  2371
## 28    28  2886
## 29    29  2470
## 30    30  1962
## 31    31  1603
## 32    32  1159
## 33    33   887
## 34    34   681
## 35    35   512
## 36    36   495
## 37    37   388
## 38    38   366
## 39    39   350
```

PRO version Are you a developer? Try out the HTML to PDF API pdfcrowd.com

```
## 40     40   246
## 41     41   226
## 42     42   288
## 43     43   265
##
## $Serology
##   time value
## 1   66   401
```

Print model

```
vbd_model
```

```
## bi_model:
## =========
##  1: model vbd {
##  2:   const p_p_risk = 1
##  3:   const p_p_immune = 0.06
##  4:   const N = 2675000
##  5:   param p_d_inc_h
##  6:   param p_d_inf_h
##  7:   param p_R0
##  8:   param p_p_rep
##  9:   param p_p_over
## 10:   param p_s_peak
## 11:   param p_s_amp
## 12:   param initI
## 13:   state S (has_output = 0)
## 14:   state E (has_output = 0)
## 15:   state I (has_output = 0)
```

PRO version Are you a developer? Try out the HTML to PDF API pdfcrowd.com

```
## 16:   state R (has_output = 0)
## 17:   state Z (has_output = 0)
## 18:   state beta_track
## 19:   state Reff
## 20:   input serology_sample
## 21:   obs Incidence
## 22:   obs Serology
## 23:   sub parameter {
## 24:     p_d_inc_h ~ truncated_gaussian(mean = 17.8/7, std = 2.3/7, lower = 0)
## 25:     p_d_inf_h ~ truncated_gaussian(mean = 4.7/7, std = 1.2/7, lower = 0)
## 26:     p_R0 ~ uniform(lower = 0, upper = 25)
## 27:     p_s_amp ~ uniform(lower = 0, upper = 1)
## 28:     p_s_peak ~ gaussian(mean = 20, std = 2)
## 29:     p_p_rep ~ uniform(lower = 0, upper = 1)
## 30:     initI ~ gamma(shape = 1, scale = 10)
## 31:     p_p_over ~ beta(1, 10)
## 32:   }
## 33:   sub initial {
## 34:     S <- max(N * (1 - p_p_immune) * p_p_risk - initI, 0)
## 35:     E <- 0
## 36:     I <- initI
## 37:     R <- N * p_p_immune * p_p_risk
## 38:     Z <- 0
## 39:     beta_track <- p_R0/p_d_inf_h * (1 + p_s_amp*cos(6.283*(-p_s_peak)/52))
## 40:     Reff <- p_R0 * S / (N * p_p_risk) * (1 + p_s_amp*cos(6.283*(-p_s_peak)/52))
## 41:   }
## 42:   sub transition {
## 43:     inline incubation_rate = 1/p_d_inc_h
## 44:     inline recovery_rate = 1/p_d_inf_h
## 45:     inline infection_rate = p_R0/p_d_inf_h
## 46:     inline transmission_rate = infection_rate*(1+p_s_amp*cos(6.283*(t_now-p_s_peak)/52))
```

PRO version Are you a developer? Try out the HTML to PDF API pdfcrowd.com

```
## 47:     beta_track <- transmission_rate
## 48:     Reff <- transmission_rate/recovery_rate * S/(N * p_p_risk)
## 49:     Z <- 0
## 50:     ode {
## 51:       dS/dt = -transmission_rate*S*I/(N*p_p_risk)
## 52:       dE/dt = +transmission_rate*S*I/(N*p_p_risk)-incubation_rate*E
## 53:       dI/dt = +incubation_rate*E-recovery_rate*I
## 54:       dR/dt = +recovery_rate*I
## 55:       dZ/dt = +incubation_rate*E
## 56:     }
## 57:   }
## 58:   sub observation {
## 59:     Incidence ~ truncated_gaussian(mean = p_p_rep * max(0, Z), std = sqrt(p_p_rep * max(
0, Z) / (1 - p_p_over)), lower=0)
## 60:     Serology ~ binomial(size = serology_sample, prob = R / (N * p_p_risk))
## 61:   }
## 62: }
```

Generate `libbi` object

```
bi <- libbi(vbd_model, input = list(serology_sample = n_serology), obs = obs,
    end_time = max(obs$Sero$time))
```

Generate prior samples (for plotting later)

```
bi_prior <- sample(bi, target = "prior", nsamples = 5000)
save_libbi(bi_prior, "salvador_prior.rds")
```

Generate and save posterior samples (after adapting the posterior distribution)

PRO version Are you a developer? Try out the HTML to PDF API pdfcrowd.com

```
bi %<>% optimise() %>% sample(proposal = "prior", nsamples = 5000) %>% adapt_proposal(min = 0.1
,
    max = 0.4) %>% sample(sample_obs = TRUE, nsamples = 5e+05, thin = 50)
save_libbi(bi, "salvador.rds")
```

Prediction

Use the posterior samples to predict up to week 80

```
pred <- predict(bi, end_time = 80, noutputs = 80, sample_obs = TRUE)
save_libbi(pred, "salvador_prediction.rds")
```

\(R_0\) calculations

Calculate \(R_0\) at the beginning of the outbreak

```
res <- bi_read(pred)
R0_calc <- copy(res[c("beta_track", "p_d_inf_h")])
R0_calc <- lapply(names(R0_calc), function(x) {
    setnames(R0_calc[[x]], "value", x)
})
R0_df <- data.table(merge(R0_calc[[1]], R0_calc[[2]]))
R0_df <- R0_df[time == 0]
R0_df <- R0_df[, `:=`(value, beta_track * p_d_inf_h)]
```

Summary statistics of \(R_0\) and the reporting rate.

```
mean(R0_df$value)
```

PRO version Are you a developer? Try out the HTML to PDF API pdfcrowd.com

```
## [1] 2.137214
```

```r
quantile(R0_df$value, c(0.025, 0.975))
```

```
##     2.5%    97.5%
## 1.787868 2.597923
```

```r
mean(res$p_p_rep$value)
```

```
## [1] 0.01990676
```

```r
quantile(res$p_p_rep$value, c(0.025, 0.975))
```

```
##       2.5%      97.5%
## 0.01761499 0.02249363
```

Plots

Predictions

Express serology as proportion immune (in results and) data

```r
res$Serology %<>% mutate(value = value/n_serology)
pred %<>% attach_file(file = "output", data = res, force = TRUE)
```

PRO version Are you a developer? Try out the HTML to PDF API pdfcrowd.com

```
pred_obs <- bi_read(pred, file = "obs")
pred_obs$Serology %<>% mutate(value = value/n_serology)
pred %<>% attach_file(file = "obs", data = pred_obs, force = TRUE)
```

Plot

```
common_plot_options <- list(x = pred, plot = FALSE, all.times = TRUE, select = list(time = 1:10
4),
    hline = c(Reff = 1), labels = c(beta_track = "beta", Reff = "R[eff]"), date.origin = as.Dat
e("2015-01-05") -
        7, date.unit = "week")
p <- list()
p[["A"]] <- do.call(plot, c(common_plot_options, list(type = "obs", obs = "Incidence")))$trajec
tories +
    scale_y_continuous("Weekly incidence") + scale_x_date("", date_labels = "%b %Y")
p[["B"]] <- do.call(plot, c(common_plot_options, list(type = "obs", obs = "Serology")))$traject
ories +
    scale_y_continuous("Percent immune", labels = percent) + scale_x_date("",
    date_labels = "%b %Y")
p[["C"]] <- do.call(plot, c(common_plot_options, list(type = "state", state = "beta_track")))$t
rajectories +
    scale_y_continuous("Transmission rate") + scale_x_date("", date_labels = "%b %Y")
p[["D"]] <- do.call(plot, c(common_plot_options, list(type = "state", state = "Reff")))$traject
ories +
    scale_y_continuous("Reproduction number") + scale_x_date("", date_labels = "%b %Y")
plot <- do.call(plot_grid, c(p, list(labels = names(p), ncol = 2)))
plot
```

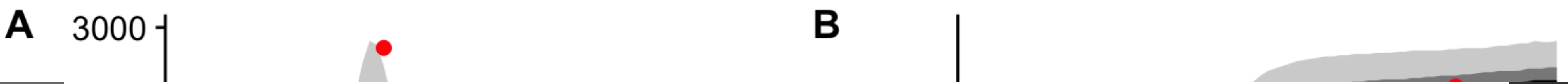

PRO version Are you a developer? Try out the HTML to PDF API pdfcrowd.com

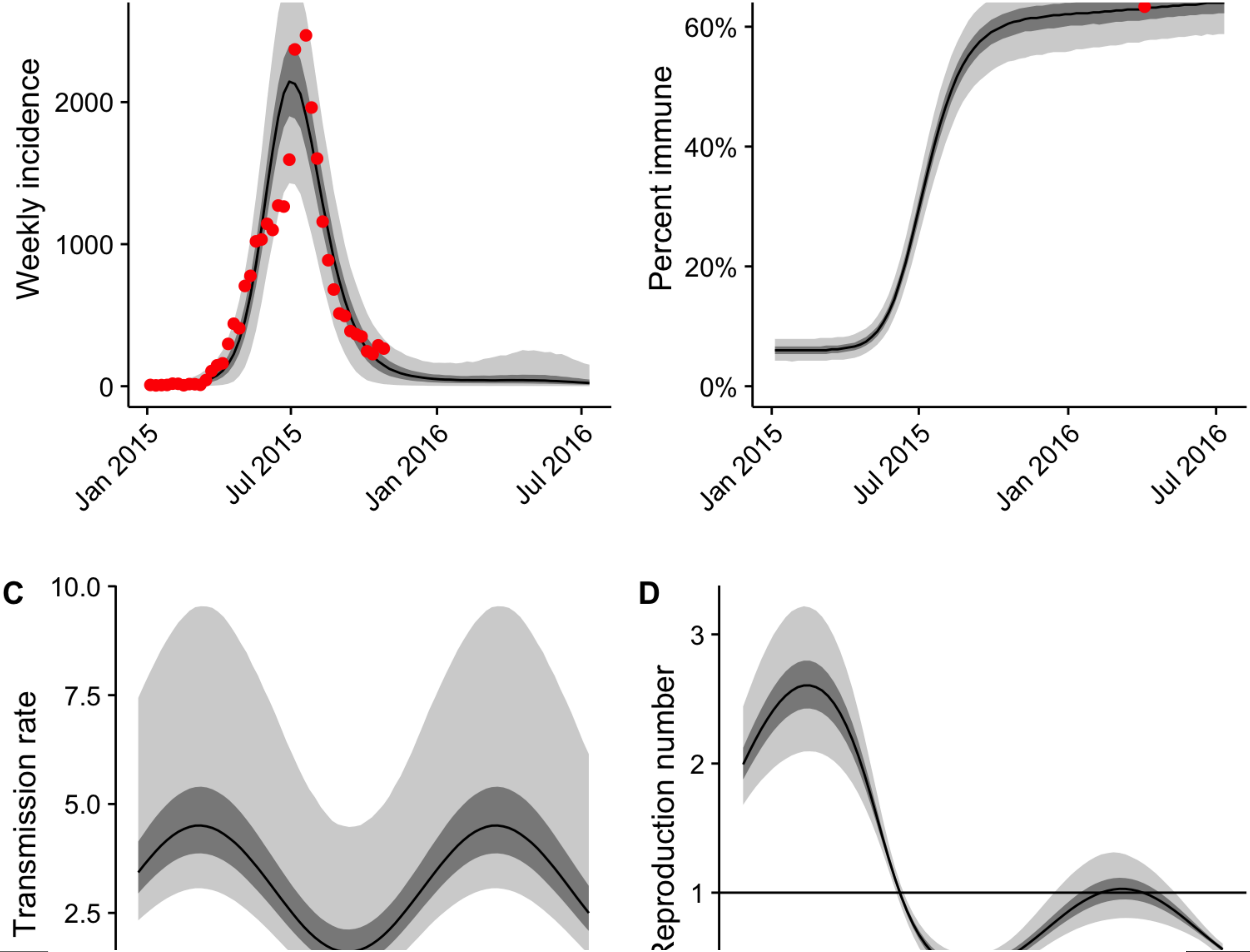

PRO version Are you a developer? Try out the HTML to PDF API pdfcrowd.com

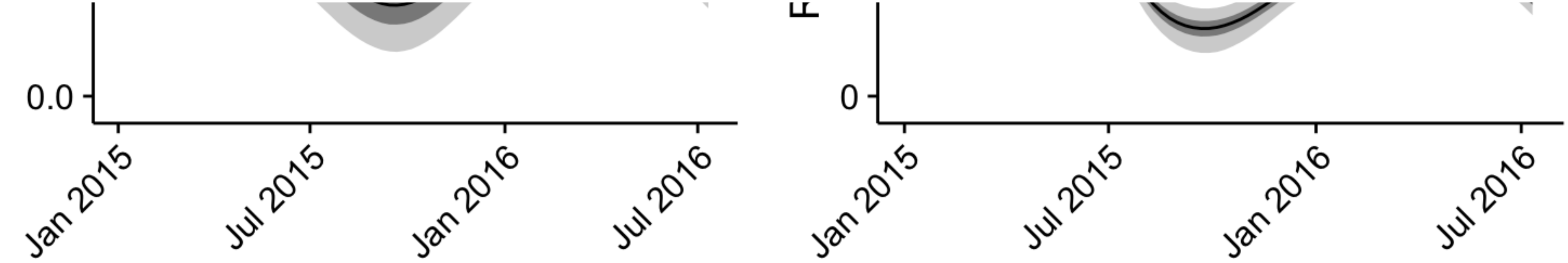

Parameter plots

\(D_\mathrm{inc}\): duration between mosquito infection caused by a human and subsequent human case to become infectious. \(D_\mathrm{inf}\): duration of human infectiousness. \(R_0\): basic reproduction number. \(r\): proportion of cases reported. \(\phi\): overdispersion of reporting. Peak week: Week of peak Zika transmission. Peak amplitude: Peak amplitude (as proportion of average transmission rate), \(I_0\): initial number of infectives.

```
p <- plot(pred, prior = bi_prior, type = c("param", "logeval"), labels = c(p_d_inc_h = "D[inc]",
    p_d_inf_h = "D[inf]", p_R0 = "R[0]", p_p_rep = "r", p_p_over = "phi", p_s_peak = "Peak~week",
    p_s_amp = "Peak~amplitude", initI = "I[0]"), pairs = FALSE, plot = FALSE)
```

Plot parameter densities

```
p$densities
```

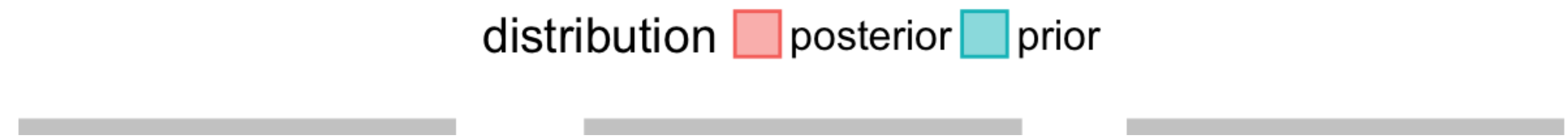

PRO version Are you a developer? Try out the HTML to PDF API pdfcrowd.com

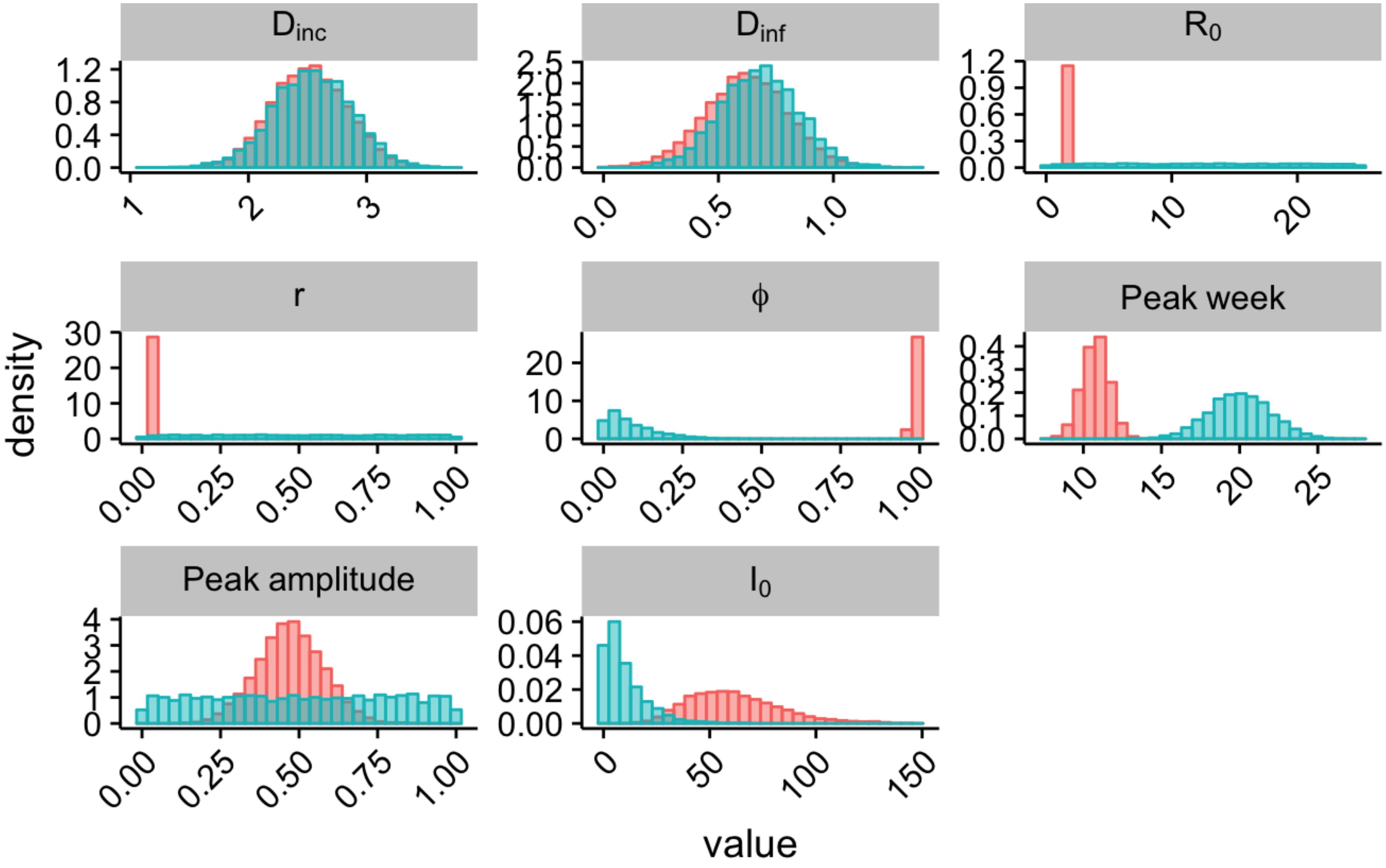

Plot parameter traces

```
p$traces
```

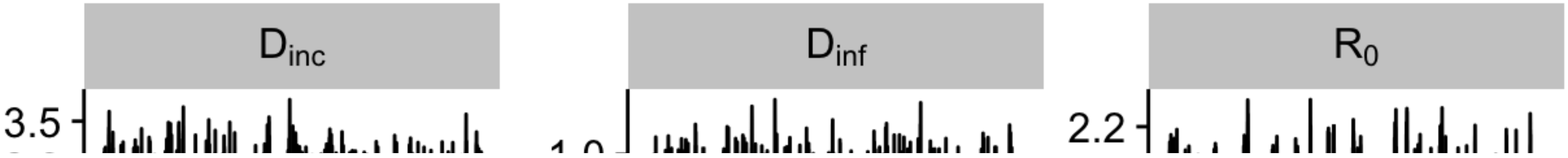

PRO version Are you a developer? Try out the HTML to PDF API pdfcrowd.com

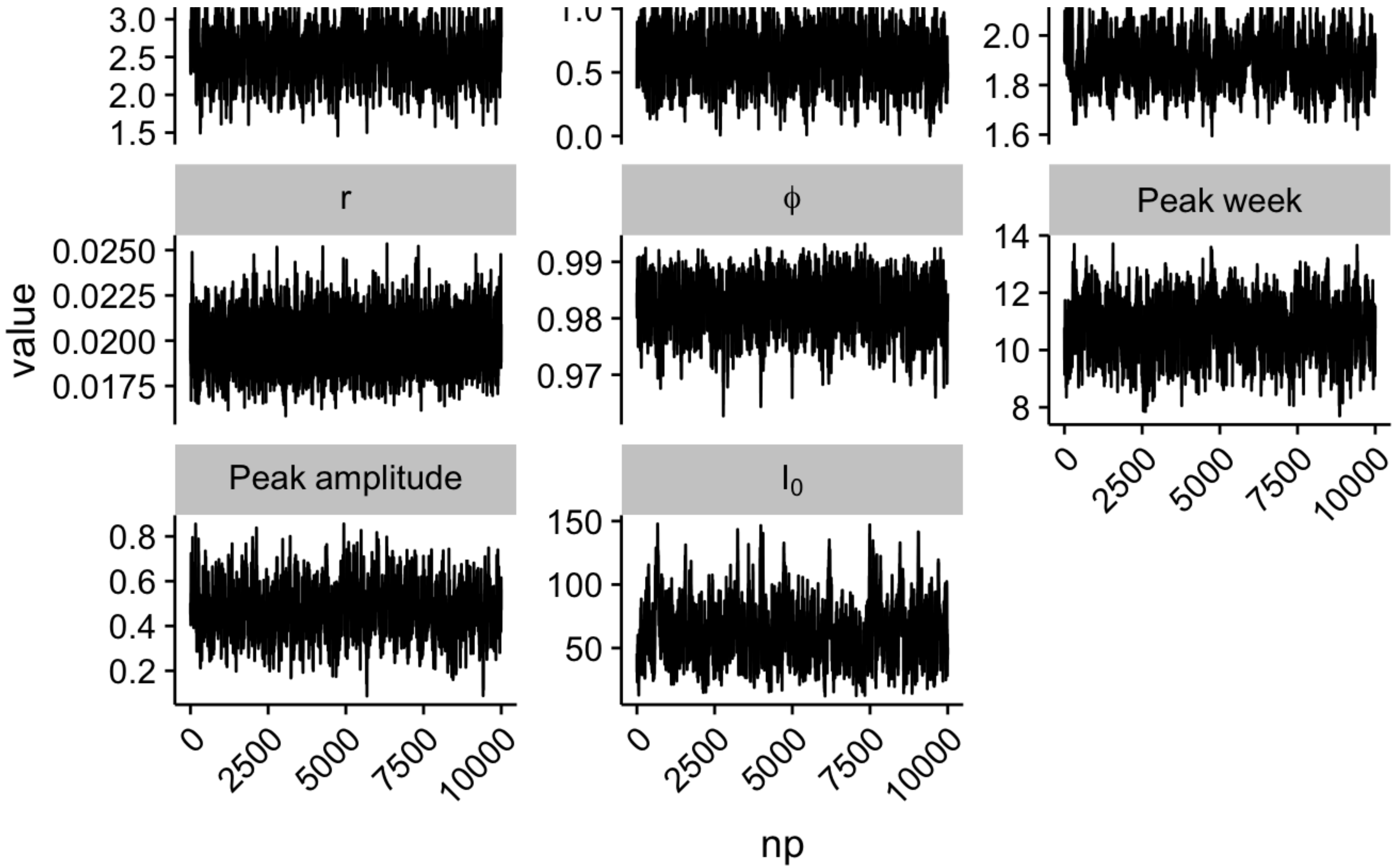

Plot paired correlations

```
p$correlations
```

I.0.

PRO version Are you a developer? Try out the HTML to PDF API pdfcrowd.com

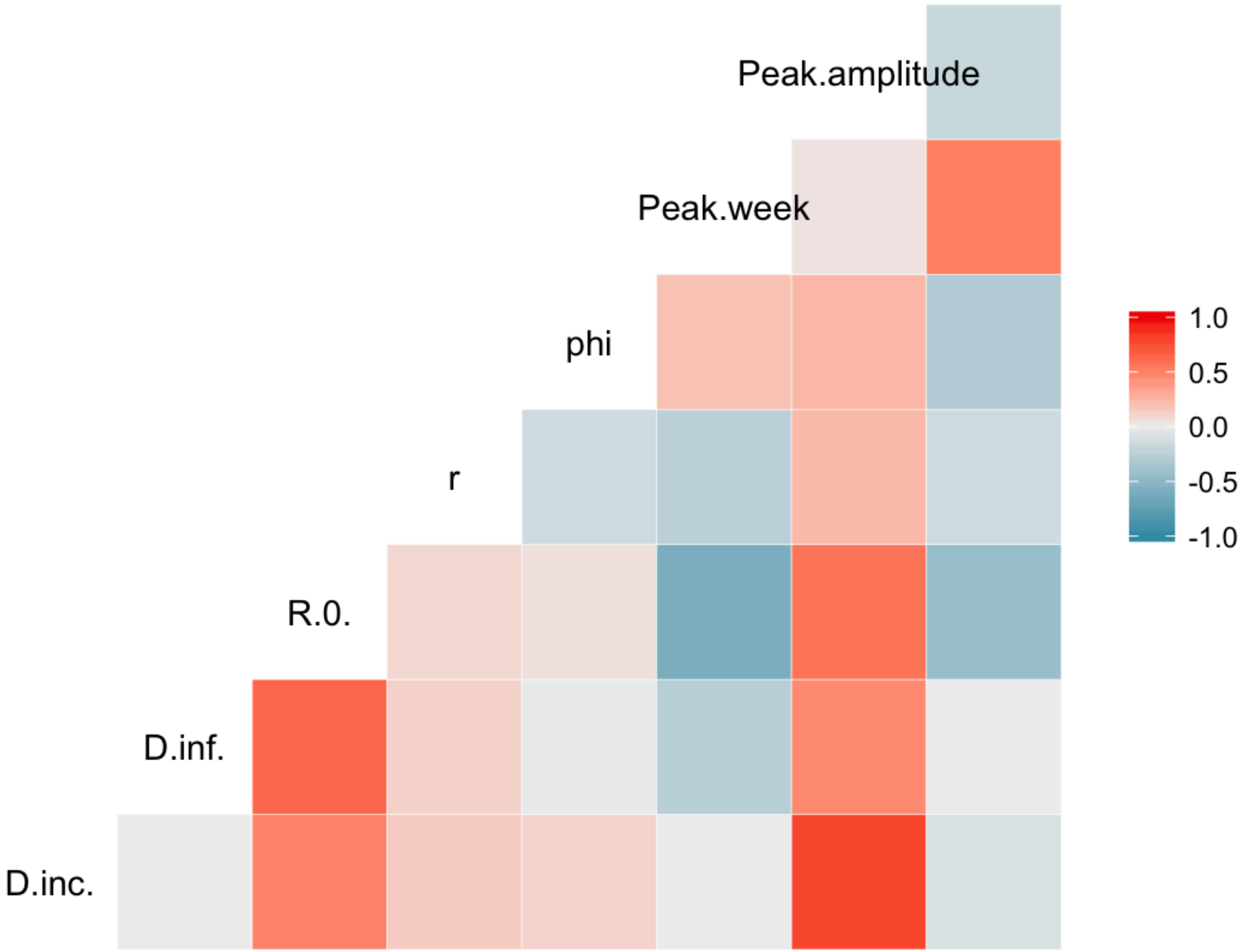

Session information

```
sessionInfo()
```

```
## R version 3.4.1 (2017-06-30)
## Platform: x86_64-apple-darwin16.6.0 (64-bit)
```

PRO version Are you a developer? Try out the HTML to PDF API pdfcrowd.com

```
## Running under: macOS Sierra 10.12.5
##
## Matrix products: default
## BLAS: /System/Library/Frameworks/Accelerate.framework/Versions/A/Frameworks/vecLib.framework
/Versions/A/libBLAS.dylib
## LAPACK: /System/Library/Frameworks/Accelerate.framework/Versions/A/Frameworks/vecLib.framewo
rk/Versions/A/libLAPACK.dylib
##
## locale:
## [1] en_GB.UTF-8/en_GB.UTF-8/en_GB.UTF-8/C/en_GB.UTF-8/en_GB.UTF-8
##
## attached base packages:
## [1] stats     graphics  grDevices datasets  utils     methods   base
##
## other attached packages:
##  [1] bindrcpp_0.2      rbi.helpers_0.2   data.table_1.10.5
##  [4] rbi_0.8.0         dplyr_0.7.2       scales_0.4.1
##  [7] cowplot_0.7.0     ggplot2_2.2.1     magrittr_1.5
## [10] rmarkdown_1.5     Defaults_1.1-1
##
## loaded via a namespace (and not attached):
##  [1] Rcpp_0.12.12       compiler_3.4.1     formatR_1.5
##  [4] RColorBrewer_1.1-2 plyr_1.8.4         bindr_0.1
##  [7] tools_3.4.1        ncdf4_1.16         digest_0.6.12
## [10] lubridate_1.6.0    evaluate_0.10      tibble_1.3.3
## [13] gtable_0.2.0       lattice_0.20-35    pkgconfig_2.0.1
## [16] rlang_0.1.1        GGally_1.3.2       yaml_2.1.14
## [19] coda_0.19-1        stringr_1.2.0      knitr_1.15.1
## [22] rprojroot_1.2      grid_3.4.1         reshape_0.8.6
## [25] glue_1.1.1         R6_2.2.2           reshape2_1.4.2
## [28] backports_1.0.5    codetools_0.2-15   htmltools_0.3.6
```

PRO version Are you a developer? Try out the HTML to PDF API pdfcrowd.com

```
## [31] assertthat_0.2.0   colorspace_1.3-2   labeling_0.3
## [34] stringi_1.1.5      lazyeval_0.2.0     munsell_0.4.3
```

PRO version Are you a developer? Try out the HTML to PDF API pdfcrowd.com
